# Novel stable QTLs identification for berry quality traits based on high-density genetic linkage map construction in table grape

**DOI:** 10.1186/s12870-020-02630-x

**Published:** 2020-09-03

**Authors:** Huiling Wang, Ailing Yan, Lei Sun, Guojun Zhang, Xiaoyue Wang, Jiancheng Ren, Haiying Xu

**Affiliations:** 1 Beijing Academy of Forestry and Pomology Sciences, Beijing, 100093 China; 2 Key Laboratory of Biology and Genetic Improvement of Horticultural Crops (North China), Ministry of Agriculture and Rural Affairs, Beijing, 100093 P.R. China; 3 Beijing Engineering Research Center for Deciduous Fruit Trees, Beijing, 100093 P.R. China

**Keywords:** Grapevine, High-density genetic linkage map, Muscat flavor, Berry firmness, Berry shape, Candidate genes

## Abstract

**Background:**

Aroma, berry firmness and berry shape are three main quality traits in table grape production, and also the important target traits in grapevine breeding. However, the information about their genetic mechanisms is limited, which results in low accuracy and efficiency of quality breeding in grapevine. Mapping and isolation of quantitative trait locus (QTLs) based on the construction of genetic linkage map is a powerful approach to decipher the genetic determinants of complex quantitative traits.

**Results:**

In the present work, a final integrated map consisting of 3411 SLAF markers on 19 linkage groups (LGs) with an average distance of 0.98 cM between adjacent markers was generated using the specific length amplified fragment sequencing (SLAF-seq) technique. A total of 9 significant stable QTLs for Muscat flavor, berry firmness and berry shape were identified on two linkage groups among the hybrids analyzed over three consecutive years from 2016 to 2018. Notably, new stable QTLs for berry firmness and berry shape were found on LG 8 respectively for the first time. Based on biological function and expression profiles of candidate genes in the major QTL regions, 3 genes (*VIT_08s0007g00440*, *VIT_08s0040g02740* and *VIT_08s0040g02350*) related to berry firmness and 3 genes (*VIT_08s0032g01110*, *VIT_08s0032g01150* and *VIT_08s0105g00200*) linked to berry shape were highlighted. Overexpression of *VIT_08s0032g01110* in transgenic *Arabidopsis* plants caused the change of pod shape.

**Conclusions:**

A new high-density genetic map with total 3411 markers was constructed with SLAF-seq technique, and thus enabled the detection of narrow interval QTLs for relevant traits in grapevine. *VIT_08s0007g00440*, *VIT_08s0040g02740* and *VIT_08s0040g02350* were found to be related to berry firmness, while *VIT_08s0032g01110*, *VIT_08s0032g01150* and *VIT_08s0105g00200* were linked to berry shape.

## Background

Marker assisted selection (MAS) technology has been widely used to improve traditional breeding accuracy and efficiency in perennial crops [1]. In recent years, one of the major objectives of grape breeding is to develop molecular markers related to traits of interest for genetic selection of target phenotypes [2]. But it needs to clarify the genetic determinisms for each given trait firstly. Quantitative trait loci (QTLs) mapping is one of the key and efficient approaches for dissecting complex traits in grapevine.

Numerous QTLs for grapevine relevant traits, including berry weight and size [3–6], sweetness and acids [7, 8], seedlessness [4, 5, 9, 10], disease-resistance traits [11–15], and so on, have been mapped. In this decade, consumers pay more and more attention on grape quality, not only the taste (flavor, texture and so on) but also the appearance traits (color, berry size and shape). Promoting berry quality traits has also been the endless pursuit of grapevine breeders. Muscat flavor, berry firmness and berry shape are three important quality traits in the breeding of new table grape varieties. Their genetic controlling mechanisms have attracted extensive attentions.

For Muscat flavor, a major QTL on linkage group (LG) 5 has been identified in three different F1 segregating progenies [16–18]. And 1-deoxy-d-xylulose-5-phosphate synthase (*Vv*DXS) has been suggested as the possible candidate gene [19]. Guo et al. [20] have investigated that berry flavor was associated with chromosome 5 too, while the significant single nucleotide polymorphisms (SNP) associated with berry flavor was identified on *VIT_205s0020g03860* (homocysteine S-methyltransferase 2). Some other QTLs with smaller effects have been found on LG1, 7, 10 and 12 [16–18, 21].

Like Muscat flavor, grape berry firmness also follows complex quantitative inheritance. That QTLs for berry firmness distributed on the different LGs has been investigated in different mapping populations. Carreño et al. [22] have firstly mapped the QTLs for berry firmness on LGs 1, 4, 5, 9, 10, 13, and 18 in ‘Muscat Hamburg’ × ‘Sugraone’ and ‘Ruby Seedless’ × ‘Moscatuel’. In the progeny of ‘Ruby Seedless’ × ‘Sultanina’, the determinants of this trait are located on LG 8 and 18 [23]. While Ban et al. [24] have found two QTLs for firmness located on LGs 3 and 10 in a ‘*V. labruscana*’ × ‘*V. vinifera*’ cross. The most recent study has reported three QTLs all detected on LG 18 in the progeny of ‘Muscat Hamburg’ and ‘Crimson Seedless’ [25]. However, most of the analyses have been performed using genetic linkage maps constructed with SSR markers, which resulted in the relatively large QTL intervals, and thus hinders the subsequent candidate genes identification.

For berry shape, to our knowledge, few studies have dealt with it in table grapes, although the diversity in berry shape is great for different grape cultivars. The wine grapes are generally round or nearly round. While, today’s cultivated table grapes have diverse shapes, which can be divided into round, nearly round, broad ellipsoid, narrow ellipsoid, ovoid, obovoid, heart-shape, cylindric and so on. However, the reason for this diversity is still unknown.

Genetic map construction is essential for the detection of QTLs associated with traits of agronomic interest. So far, a series of parental and consensus genetic maps have been developed by applying amplified fragment length polymorphism (AFLP) [26], sequence related amplified polymorphism (SRAP) [27], and single sequence repeat (SSR) [28, 29] markers to different bi-parental segregating populations in grapevine. But majority of the genetic maps consist a limited number of markers with large map spacing and low resolution, thus are not able to provide precise and complete information about the numbers and locations of QTLs controlling the traits [30]. With the rapid development of the next generation sequencing (NGS) technology, genotyping through sequencing becomes the most direct and powerful method for large-scale detection of single nucleotide polymorphism (SNP), which are considered as the markers of best choice for high-density genetic map construction [31, 32]. At present, several high-density genetic maps have been constructed using NGS technologies, and some new QTLs have been successfully identified in grapevine [7, 11, 33, 34]. There is no doubt that these high-density maps improve the efficiency and accuracy of QTL mapping, which can be used more effectively in breeding programs. However, QTL analysis for targeted traits mainly depends on the polymorphism between parents of the mapping populations. Lots of markers have distinct genotypes or linkage relationships in different hybrid populations [35]. Therefore, it is necessary to develop new high-density genetic linkage maps of grapevine for further QTL analysis of interest traits in grapevine.

Among all the NGS strategies, whole genome re-sequencing can identify whole genome wide differences between individuals and a large number of SNP markers [36]. But it is still too costly to apply in multiple samples, and generally unnecessary for linkage mapping. Specific length amplified fragment sequencing (SLAF-seq), a reduced representation sequencing approach, has been developed, and exhibits advantages in large-scale de novo SNP discovery and genotyping [37]. In the last 5 years, a series of high-density genetic maps have been constructed based on SLAF-seq in various plant species [38, 39]. Guo et al. [40] and Wang et al. [41] have successfully applied this method to construct high-density genetic maps in grapevine.

On these bases, SLAF-seq was currently used in whole-genome genotyping for grapevine F1 lines; a consensus high-density genetic map was constructed with the developed SLAF markers. The map will facilitate the further precise identification of QTLs for major agronomic traits, and marker assisted selection in grape breeding programs. Moreover, the berry Muscat flavor, berry firmness and berry shape of F1 progenies and two parents were analyzed for 2–3 successive years. QTLs for these three traits were identified and analyzed. The candidate genes were predicted and validated by real time PCR and transgenic Arabidopsis. The results will broaden our understanding of the genetic control of these fruit traits, the tightly linked markers would be used to improve the fruit quality of grape.

## Methods

### Plant materials

The mapping population used in this study (line 1002; *n* = 160) was generated by crossing ‘Moldova’ (*V. vinifera* × *V. labruscana*) and ‘Ruidu Xiangyu’ (*V. vinifera*) [42] in 2010. The female parent ‘Ruidu Xiangyu’ was selected from a cross of ‘Jingxiu’ × ‘Xiangfei’ (Both are local varieties in China). ‘Ruidu Xiangyu’ is a grape with ovoid shape, greenish-yellow color, firm and crisp flesh and an excellent Muscat flavor. While the male parent ‘Moldova’ is a grape with ellipsoid shape, dark blue skin, soft flesh and neutral flavor. It was derived from a cross of ‘Guzal Kara’ and ‘Villard blanc’ in the Republic of Moldova in the 1960s and introduced to China in 1997. Moreover, ‘Moldova’ has high resistance to downy mildew and grey mold [43]. Taken together, there are great genetic differences between two parents, which supply excellent materials for high-density genetic map construction and subsequent QTL mapping. The plants of the two parents and their progeny were grown on their own roots in the experimental vineyard at Beijing academy of Forestry and Pomology Sciences (39°58′ N and 116°13′ E). The grapevines were north-south oriented, and the space of plantation was 1.0 m × 2.5 m. They were maintained under routine cultivation conditions as described by the method of Chen et al. [7].

### Phenotypic measurements

Fully ripened berries (°Brix≥18) from F1 individuals and the two parents were collected in three successive seasons (2016–2018). Due to mechanical injury (In Beijing, the grape vine has to be buried underground to protect it from cold in winter), poor fruit setting, or bunch rot, not all F1 plants can set fruit or produce enough fruits for the later experiments. The number of harvested samples varied every year. In total, 115 genotypes were used in 2016, 133 in 2017, and 123 in 2018 for phenotypic measurement.

Muscat flavor was scored according to the previous method [16] with little modification. The intensity of Muscat flavor was scored using 4-point scale (0: no flavor, 1: light Muscat flavor, 3: medium-level Muscat flavor, 5: strong Muscat flavor). Three berries per offspring were tasted by five tasters independently. The average value was used for the later analysis.

Berry firmness was assessed over 2 years (2017–2018) according to the method of Carreño et al. [22] with universal TA. XTplus testing machine (Stable Micro Systems, Godalming, Surrey, UK). By using the device, firmness values were expressed as force (N) required for a 20% deformation of the berries. The average value of 20 berries per offspring was used in subsequent analysis.

The berry shape index (ShI) was determined as the ratio of the berry length (BL) to the berry diameter (BD). Berry length and diameter were assessed according to the OIV descriptors (OIV, 2009) with little modification. Thirty berries taken from the middle part of five representative clusters were measured respectively. All measurements were performed with a hand caliper. The mean value of 30 berries per offspring was used in further analysis.

### Statistical analysis

Sigmaplot 10.0 software was used to check the frequency distribution of phenotypic data. The mean, minimum, maximum and coefficient of variation (CV) of the phenotypic data were analyzed by the SPSS 13.0 software (SPSS, United States). The broad sense heritability (*H*_*b*_^*2*^) was estimated following the methods of Liu et al. [44]. The formula is *H*_*b*_^*2*^ = V_g_ / (V_g_ + V_e_); Vg = (V_f_ - V_e_) / 3, where *H*_*b*_^*2*^ is the broad sense heritability, V_g_ is the genetic variance, V_f_ is the mean variance between individuals, V_e_ is the mean variance within individuals.

### DNA isolation

For DNA isolation, young and healthy leaves were harvested from each individual F1 plant and the two parents at the beginning of the vegetative period. Samples were immediately frozen in liquid nitrogen and stored in a − 70 °C freezer for further analysis.

DNA was extracted after grinding the samples to a fine powder with the Mixer-Mill-MM 300 grinder (Retsch, Haan, Germany) using the DNeasy plant mini prep kit (Qiagen). DNA concentration was measured by a NanoDrop spectrophotometer (ND2000, Thermo Fisher Scientific, USA). The integrity of DNA was checked by gel electrophoresis.

### SLAF library construction and sequencing

SLAF-seq libraries were constructed as described by Sun et al. [37] with a small modification. In brief, a pilot SLAF experiment was performed to optimize conditions for obtaining maximum SLAF-seq efficiency. Based on the results, two enzymes, *RsaI* and *HaeIII* (New England Biolabs, USA), were used to digest the genomic DNA of each sample. The digested fragments were purified and added with a single nucleotide (A) overhang. After that, the duplex tag-labeled sequencing adapters (PAGE-purified, Life Technologies, USA) were ligated to the A-tailed fragments. Then the diluted restriction-ligation DNA samples, dNTP, Taq DNA polymerase (NEB), and PCR primers were used for PCR reaction. The PCR products were purified, and 48 DNAs were pooled. The pooled DNAs were separated on a 2% agarose gel. With a Gel Extraction Kit (Qiagen, Hilden, Germany), the fragments ranging from 400 to 450 bp (with indexes and adaptors) in size were obtained. Thereafter, the Gel-purified products were diluted, and pair-end sequencing (150 bp from each end) was performed on an Illumina HiSeq 2500 system (Illumina, Inc., San Diego, CA, USA). Real-time monitoring was performed for each cycle during sequencing, the ratio of high quality reads (quality score > 30) in the raw reads and GC content were calculated for quality control.

### Sequencing data grouping and genotyping

The procedures of sequencing data grouping and genotyping were performed as described previously [37]. Briefly, the Burrows-Wheeler Aligner (BWA) software was used to align clean reads from each sample against the *Vitis vinifera* reference genome (ftp://ftp.ensemblgenomes.org/pub/plants/release-25/fasta/vitis_vinifera/) with default parameters. Sequences mapped to the same position were defined as a single SLAF locus. Locus with two to four SLAF tags was identified as polymorphic SLAF. There was SNP or indel difference between the sequences of SLAF tags at the same locus. The average sequence depths of SLAF markers were greater than 20-fold in parents and greater than 8-fold in progeny. According to the genotype of parents, the SLAF tags were encoded and grouped into eight segregation patterns (aa × bb, ab × cc, ab × cd, ef × eg, hk × hk, lm × ll, nn × np and cc × ab). Because of the population type of F1, the markers with segregation patterns of aa × bb were excluded for genetic map construction. In order to ensure the quality of the genetic map, three stringent filtering criteria were considered for the SLAF markers: i) with the average sequence depths of > 40-fold in the parents; ii) The number of SNP is < 8 per SLAF marker; iii) with less than 5% missing data in F1 populations. In addition, the chi-square test was then performed to examine the segregation distortion, and markers with significant segregation distortion (*P* < 0.01) were initially excluded from map construction.

### Genetic linkage map construction

Based on the locations on the grape genome, the filtered SLAF markers were partitioned into 19 linkage groups. The modified logarithm of odds (MLOD) scores between markers was calculated, and markers with MLOD scores < 5 were filtered out before ordering. Highmap software was used to construct the genetic map of each linkage group as described by Liu et al. [45]. The error correction strategy of SMOOTH [46] was applied to correct genotyping errors, and a *k*-nearest neighbor algorithm [47] was used to impute genotyping missing. The enhanced algorithm of Gibbs sampling, spatial sampling and simulated annealing (GSS) [48, 49] were employed to order markers. Map distances in centi-Morgans (cM) were calculated using the Kosambi mapping function [50]. For the construction of the consensus map, markers mapped in both parental maps and heterozygous markers (ab × ab) were used. Finally, the haplotype map, the heat map, and collinearity between the genetic and physical positions were analyzed by the method of Liu et al. [45] to evaluate the quality of the constructed linkage map.

### QTL mapping

Quantitative trait loci (QTLs) analysis for the berry Muscat flavor, berry firmness and berry shape were carried out on the consensus and parental maps using interval mapping with MapQTL 6.0 [51]. The threshold of LOD scores for evaluating the statistical significance of QTL effects was determined using 1000 permutations. Based on these permutations, the LOD threshold for “berry shape” was set at 5 corresponding to 99% confidence interval in all the 3 years; while for “berry firmness” and “Muscat flavor”, the threshold value were both set to 3.0 at 95% confidence interval.

The genes within QTLs were identified by mapping the associated markers on the physical map. The genes were annotated and analyzed via the databases of Ensembl Plants (http://plants.ensembl.org/index.html) and NCBI (https://www.ncbi.nlm.nih.gov/). Possible candidate genes related to a specific trait were predicted based on their biological functions.

### Validation of candidate genes

To further validate the candidate genes related to each specific trait, grape berries of two parent cultivars, ‘Moldova’ and ‘Ruidu Xiangyu’, were sampled at young grape berry stage (stage A, pea-size berries), veraison (stage B, 50% berries turning red or soft), and fully ripening stage (stage C, °Brix ≥18) in 2018. The Muscat flavor, berry firmness, ShI and candidate gene expression of the sampled berries were analyzed.

For RNA extraction, three biological replicates were collected for each sample. For each biological replicate, 10 berries without seeds were ground into powder and 1 g powder was used. Total RNA was isolated from the different samples using a Plant RNA Isolation Kit (Sigma RT-50, St. Louis, MO, USA). The RNA integrity was verified using agarose gel electrophoresis. The first-strand complementary DNAs (cDNAs) were synthesized according to the manufacturer’s instruction of AMV reverse transcriptase (Promega A3500). Then, two-step qPCR was carried out in the CFX 96 RT-PCR system (Bio-Rad, Richmond, CA) using a SYBR PCR kit (Tiangen, Beijing, China). The primers for all the candidate genes were designed by the Primer-Blast from NCBI (https://www.ncbi.nlm.nih.gov/tools/primerblast/) and their sequences are listed in Supplementary Table 1. Both *VvGAPDH* (CB975242) and *Vvubiquitin* (EC929411) were used as reference genes. The q-PCR reaction for each biological replicate was carried out in triplicate times.

### Transformation of *VIT_08s0032g01110* in Arabidopsis

The full-length cDNA of *VIT_08s0032g01110* were synthesized in the Beijing Sunbiotech Company (Beijing, China). The CDS of *VIT_08s0032g01110* was then fused to the downstream of CaMV35S promoter at the *Bgl II* (5′ end)/ *BstE II* (3′ end) sites by substitution of the GUS gene in pCAMBIA1301 vector. Lines of transgenic *Arabidopsis* with the *pCAMBIA1301:VIT_08s0032g01110* construct were obtained through the infection of inflorescence by *Agrobacterium tumefaciens* strain GV3101. *Arabidopsis* Columbia-0 ecotype was deposited at the Key Laboratory of Biology and Genetic Improvement of Horticultural Crops (North China), Ministry of Agriculture and Rural Affairs in Beijing. The Positive transgenic Arabidopsis lines were screened in the presence of Hygromycin (Roche, Germany), and PCR was performed to select T1 plants.

## Results

### Phenotypic data analysis

**Fig. 1:**
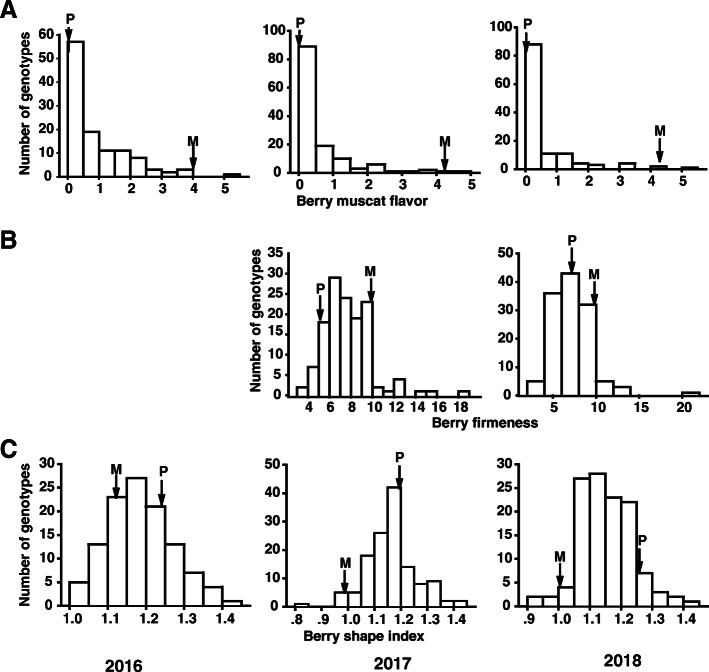
Phenotypic distribution of Muscat flavor (**a**), berry firmness (**b**) and Berry shape index (**c**) over 2–3 years for ‘Moldova’ × ‘Ruidu Xiangyu’ progeny. The plot was based on mean values of each genotype. The parental mean values are indicated by P (paternal) and M (maternal)

The phenotypic variation ranges of Muscat flavor (MF), berry firmness (BF) and berry shape index (ShI) for the two parents and the F1 progenies were presented in Table 1 and Supplementary Table 2. As the results shown, the berries of ‘Ruidu Xiangyu’ (female parent) with pronounced Muscat flavor showed a high Muscat flavor score. No Muscat flavor was tested in the berries of male parent ‘Moldova’. In F1 population, the distribution for Muscat flavor score was continuous but highly skewed towards low values. Distributions of Muscat flavor score for each individual year are presented in skewed distribution as given in Fig. 1a.

**Table 1:** An overview of the phenopypic data of F1 population and two parents for each trait

Trait	Year	Ruidu Xiangyu (Female parent)	Moldova (Male parent)	Mid-Parent value	F1 population			
					Mean	Range of variation	CV%	***Hb***^***2***^ (%)
MF	2016	4.00	0.00	2.00	0.86	0.00–5.00	118.02	70.98
	2017	4.25	0.00	2.13	0.55	0.00–4.50	158.07	58.85
	2018	4.20	0.00	2.10	0.47	0.00–5.00	196.08	65.18
BF (N)	2016	9.69	5.15	7.42	7.68	3.46–18.71	29.46	64.46
	2017	9.82	7.54	8.68	7.18	2.57–21.81	32.67	66.98
	2018	–	–	–	–	–	–	–
ShI	2016	1.12	1.24	1.12	1.19	1.02–1.45	7.09	61.91
	2017	1.00	1.18	1.00	1.16	0.83–1.44	8.05	69.32
	2018	1.03	1.26	1.03	1.15	0.92–1.44	7.79	66.32

Note: CV indicates coefficient of variation; *H*_*b*_^*2*^ represents the broad sense heritability; MF is abbreviation of Muscat flavor; BF is abbreviation of berry firmness; ShI represents berry shape index

Berry firmness was accessed in 2017 and 2018. The berries of ‘Ruidu Xiangyu’ and ‘Moldova’ showed medium and soft firmness, respectively, with mean force values of 9.69 and 5.15 N in 2017, and 9.82 and 7.54 N in 2018. In their progenies, the average of BF in 2017 and 2018 ranged from 3.46 to 18.71, 2.57 and 21.81 N respectively. Extreme phenotypes with higher or lower values than those of the parents were investigated, indicating transgressive segregation existed in the F1 progenies. The frequency distributions of BF over 2 years were shown in a normal distribution (Fig. 1b).

In terms of berry shape, the berries of ‘Ruidu Xiangyu’ were nearly rounded; ‘Moldova’ berries showed elliptical shape. Higher ShI value was observed in ‘Moldova’. The values of ShI in F1 population showed continuous variation, and transgressive distribution was observed (Fig. 1c). The mean value of ShI in F1 population was 1.19 (2016), 1.16 (2017), and 1.15 (2018). The means of ShI in F1 population were over or equal to the mid-parent value in three successive years. Approximately similar normal phenotypic data distributions of ShI were examined for all 3 years (Fig. 1c).

As phenotypic data shown, all three traits showed quantitative inheritance, suggesting that they were controlled by multiple genes. However, high broad-sense heritability (*H*_*b*_^*2*^) (more than 50%) was investigated for each trait (Table 1), which further indicated that there might be major QTLs affecting phenotypes.

### Construction of high-density genetic map

After SLAF library construction and high-throughput sequencing, a total of 310.67 M paired-end reads was generated. The Q30 (a quality score of at least 30, indicating a 0.1% chance of an error, and thus 99.9% confidence) ratio was 88.97% and the average guanine-cytosine (GC) content was 39.57%. The reads were then mapped to the reference grapevine genome, a total of 263,676 high-quality SLAF tags were detected. The numbers of SLAFs in the male and female parents were 184,657 and 185,166, respectively. Among the detected 263,676 high-quality SLAF tags, 96,416 were polymorphic with a polymorphism ratio of 36.57%. Of these polymorphic SLAFs, 61,477 were classified into eight segregation patterns (Supplementary Figure 1). Except for the aa × bb genotype, the other patterns were used for genetic map construction. In final, 6436 SLAF markers were suitable for genetic map construction. After screening out the redundant markers at same genetic location, total 3411 SLAF markers (1563 lm × ll, 1277 nn × np, 293 hk × hk, 246 ef × eg and 32 ab × cd) (Supplementary Table 3) were used for the final consensus high-density linkage map construction.

**Fig. 2:**
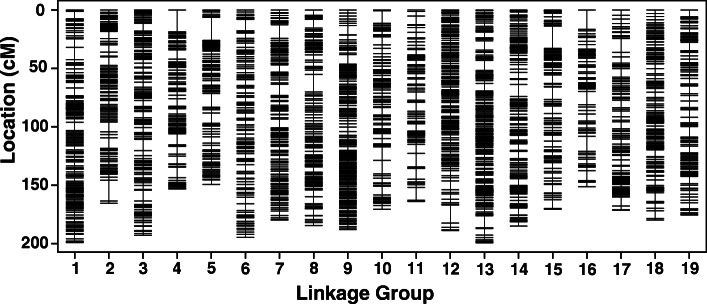
Genetic map lengths and marker distribution in 19 linkage groups of the consensus map. Genetic distance is indicated by the vertical scale in centi-Morgans (cM). Black lines represent mapped markers. 1–19 represent corresponding linkage groups ID

After linkage analysis, 3411 SLAF markers were clustered on 19 linkage groups (LG1-LG19), which were numbered according to the chromosome numbers (Fig. 2). As shown in Supplementary Table 4, there were 2134 SLAF markers in the map of ‘Moldova’ (*V. vinifera* × *V. labruscana*) with total length 3342.75 cM. The average distance between adjacent markers was 1.57 cM. The length of each LG ranged from 135.7 cM (LG9) to 222.12 cM (LG10). LG15 contained only 47 SLAF markers with an average marker interval of 3.51 cM, whereas LG 13 contained the most markers (188) with an average marker interval length of 1.06 cM. The percentage of “Gap < 5 cM” which reflected the degree of linkage between markers ranged from 86% (LG15) to 98% (LG18–19).

The maternal map of ‘Ruidu Xiangyu’ (*V. vinifera*) included 1848 SLAF markers. This map encompassed 3018.9 cM, with an average distance between adjacent markers of 1.63 cM. The largest LG was LG14 with 159 SLAF markers and an average interval length of 1.08 cM. The shortest LG17 contained 65 markers with an average interval length of 1.60 cM. The percentage of the intervals between adjacent markers less than 5 cM ranged from 82 to 97% (Supplementary Table 5).

The consensus grape map included 3411 markers with a total genetic distance of 3365.41 cM (Table 2, Fig. 2 and Supplementary Figure 2). The average interval distance between markers was 0.98 cM. The genetic length of the LGs ranged from 149.4 cM (LG5) to 199.44 cM (LG13). LG14 contained the highest number of markers (262), spanning 185.06 cM with the average genetic distance of 0.71 cM, whereas LG11 was the least saturated with the length of 164.15 cM and contained the lowest number of markers (only 103). The percentage of “Gap < 5 cM” in each LG was more than 92% with the average value up to 97%. The largest gap was located in LG15 with 18.98 cM in length on this map.

**Table 2:** The information of the consencus high-density genetic map

Chr ID	Genome Size (Mb)	No of SLAFs	Distance (cM)	Average distance between markers (cM)	Largest gap	Gap<5 cM	Kb/cM
Chr1	25.31	176	199.22	1.13	10.34	97%	127.04
Chr2	20.13	151	165.57	1.09	17.83	96%	121.61
Chr3	22.05	179	192.96	1.07	9.61	97%	114.25
Chr4	25.67	131	153.37	1.17	18.74	95%	167.38
Chr5	27.28	146	149.4	1.02	19.65	97%	182.58
Chr6	23.06	185	194.71	1.05	9.75	98%	118.43
Chr7	24.09	179	179.96	1.01	11.51	98%	133.89
Chr8	24.00	215	184.69	0.86	15.06	98%	129.95
Chr9	25.19	254	188	0.74	8.03	99%	133.98
Chr10	20.30	160	170.63	1.07	11.69	96%	118.95
Chr11	21.55	103	164.15	1.59	11.62	92%	131.29
Chr12	26.02	248	188.96	0.76	15.48	99%	137.70
Chr13	29.66	261	199.44	0.76	10.22	98%	148.72
Chr14	32.46	262	185.06	0.71	10.26	98%	175.39
Chr15	21.77	126	170.59	1.35	18.98	94%	127.61
Chr16	24.44	119	151.43	1.27	16.57	96%	161.38
Chr17	19.25	140	171.6	1.23	11.90	96%	112.19
Chr18	37.02	187	179.97	0.96	7.40	98%	205.70
Chr19	25.75	189	175.7	0.93	9.09	95%	146.58
Total	475.00	3411	3365.41	0.98	/	97%	141.14

### Evaluation of the high-density genetic linkage maps

The quality of the constructed genetic map is closely related to the accuracy of subsequent QTL mapping. Here, the sequence depths of SLAF markers on the map were analyzed firstly. As the results shown, the average sequencing depths of these 3411 markers were 58.33-fold for ‘Moldova’, 75.13-fold for ‘Ruidu Xiangyu’, and 18.25-fold for each individual progeny. The numbers of SLAF markers in each individual ranged from 3196 to 3411 with an average of 3389, and the sequencing depth ranged from 9.12-fold to 30.54-fold (Supplementary Figure 3). These analysis results reflected the validity of molecular markers genotyping to a certain extent.

It is believed that haplotype and heat maps can directly reflect the quality of the genetic maps. Haplotype maps show recombination events in individuals, and heat maps reflect the recombination frequency and mapping location between markers. A haplotype map for LGs of the consensus map was shown in Supplementary Figure 4. As the results shown, the occurrence of double crossovers and deletion ratio were low, indicating genotyping and marker-order of the LGs were accurate and reliable.

Heat maps were generated by using pair-wise recombination values for all the mapped SLAF markers. The heat maps for the paternal were also shown in Supplementary Figure 5. The linkage between markers decreases with the increase of genetic distance, which indicates that the order of markers in the LGs is correct.

Furthermore, the collinearity between the genetic and physical positions on a linkage map was also analyzed. A high level of genetic collinearity was observed between 19 LGs and the reference genome (Supplementary Figure 6). As shown in Supplementary Table 6, the Spearman correlation coefficient ranged from 0.85 to 0.98, and it was higher than 0.90 in most LGs.

In general, from the results of haplotype maps, heat maps and collinearity analysis, the genetic maps constructed were of good performance for further QTL analysis.

### QTL identification

QTL analyses were performed using both the consensus and parental genetic maps. The QTLs detected for all the three traits are summarized in Table 3 based on the consensus map. A total of 9 stable QTLs were mapped on the consensus genetic map using the interval mapping method. Of the 9 QTLs, four contributed to berry Muscat flavor, two were associated with fruit firmness, and the remaining three were related to berry shape. Compared to those mapped on consensus map, the QTLs with same flanking markers were detected using parental genetic maps (Supplementary Table 7–8 and Supplementary Figure 7).

**Table 3:** Summary of QTLs based on consensus map for three berry related traits over 3 successive years

Trait	QTL	Chr	Year of detection	Flanking Markers	Interval (cM)	Maximum LOD	PVE (%)
MF	qMF-1	5	2016	Marker2668298- Marker2755502	30.802–35.572	3.58	14.40
	qMF-2	5	2016	Marker2793749- Marker2853250-	39.685–43.448	4.22	16.80
	qMF-3	5	2017	Marker2668298-Marker2853250	30.802–43.448	7.19	21.80
	qMF-4	5	2018	Marker2761785-Marker2754215	27.248–44.108	3.71	19.70
BF	qBF-1	8	2017	Marker1558508-Marker1505260	150.415–154.437	4.14	19.90
	qBF-2	8	2018	Marker1558508-Marker1442546	150.415–154.123	3.13	20.10
ShI	qShI-1	8	2016	Marker1415438- Marker1563052	2.565–8.655	6.45	20.50
	qShI-2	8	2016	Marker1399465	24.648	5.23	16.50
	qShI-1	8	2017	Marker1415438-Marker1563052	2.565–8.655	5.59	19.10
	qShI-3	8	2018	Marker1529171-Marker1456093	0–36.002	5.60	25.00

Note: Chr indicates chromosome; LOD indicates the logarithm of odds score; PVE indicates the phenotypic variance explained by individual QTL; MF is abbreviation of Muscat flavor; BF is abbreviation of berry firmness; ShI represents berry shape index

**Fig. 3:**
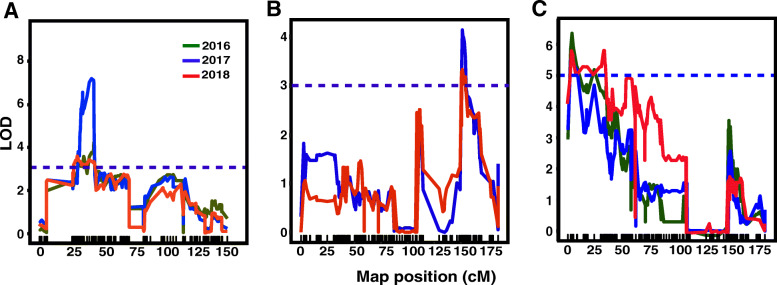
Precise locations of major QTLs for Muscat flavor, berry firmness and berry shape in the consensus map. **a** LOD curves of QTL mapping for Muscat flavor on chromosome 5 in 2016–2018. **b** LOD curves of QTL mapping for berry firmness on chromosome 8 in 2017–2018. **c** LOD curves of QTL mapping for berry shape index on chromosome 8 in 3 successive years. Short lines on x-axis indicate the genetic positions of the SLAF markers. Dashed dots showed the threshold of LOD

Four QTLs controlling Muscat flavor score were found on LG5 in 3 successive years, respectively. The phenotypic variance explained by individual QTL (PVE) ranged from 14.40 to 21.80%. In 2016, qMF-1 (maximum LOD = 3.58; 14.40% of PVE) and qMF-2 (maximum LOD = 4.22; 16.80% of PVE) were detected. In 2017, qMF-3 (maximum LOD = 7.19; 21.80% of PVE) was mapped covering 30.802–43.448 genetic interval. qMF-4 (maximum LOD = 3.71; 19.70% of PVE) was identified in 2018. The four QTLs covered the same genetic interval (30.802–35.572 cM and 39.685–43.448 cM) respectively (Table 3 and Fig. 3a).

Two significant stable QTLs linked to berry firmness were both located on LG8 (qBF-1 and qBF-2) (Table 3). In 2017, the QTL of qBF-1 was identified with a maximum LOD score of 4.14 and an 19.90% of PVE. qBF-2 was detected in 2018, which explained 20.10% of PVE. qBF-1 and qBF-2 shared the same genetic interval 150.415–154.123 cM. The position of the two QTL peaks was steady across the two seasons (Fig. 3b).

There were three QTLs linked to berry shape located on LG8, including qShI-1 (maximum LOD = 6.45, 20.50% of PVE, in 2016; maximum LOD = 5.59, 19.10% of PVE in 2017), qShI-2 (maximum LOD = 5.23, 16.50% of PVE), and qShI-3 (maximum LOD = 5.60, 25.0% of PVE). The QTLs of qShI-1 and qShI-3 covered the stable genetic interval (2.565–8.655 cM) between Marker1415438 and Marker1563052 in 3 successive years (Fig. 3c).

### Candidate genes involved in berry quality traits

Because stable genetic intervals were detected for each trait across years, the candidate genes located within these common genetic intervals were henceforward being focused on. The linked markers in the confidence intervals were mapped on to the grapevine reference genome sequence. Four genomic regions of 2.90–4.11 Mb of chromosome 5 (related to Muscat flavor), 4.51–6.26 Mb of chromosome 5 (related to Muscat flavor), 13.44–15.71 Mb of chromosome 8 (linked to berry firmness), and 4.33–9.56 Mb of chromosome 8 (linked to berry shape index) were further analyzed. 157, 153, 244 and 141 genes in these regions were identified and annotated, respectively. Based on their biological function, 3, 3, 10 and 11 genes, respectively, were highlighted as good candidates for each trait (Supplementary Table 9). For Muscat flavor, a probable 1-deoxy-D-xylulose-5-phosphate synthase (*VIT_05s0020g02130*) was found in the region of 2.90–4.11 Mb of chromosome 5. A predicted expansin-A6 (*VIT_08s0007g00440*) and a probable pectate lyase 4 (*VIT_08s0040g02740*) related to berry firmness were found in the region 13.44–15.71 Mb of chromosome 8. Additionally, *VIT_08s0032g01110* (predicted axial regulator YABBY 5) was included in the 11 good candidate genes related to the berry shape index.

### Analysis of expressions of candidate genes during grape berry development

**Fig. 4:**
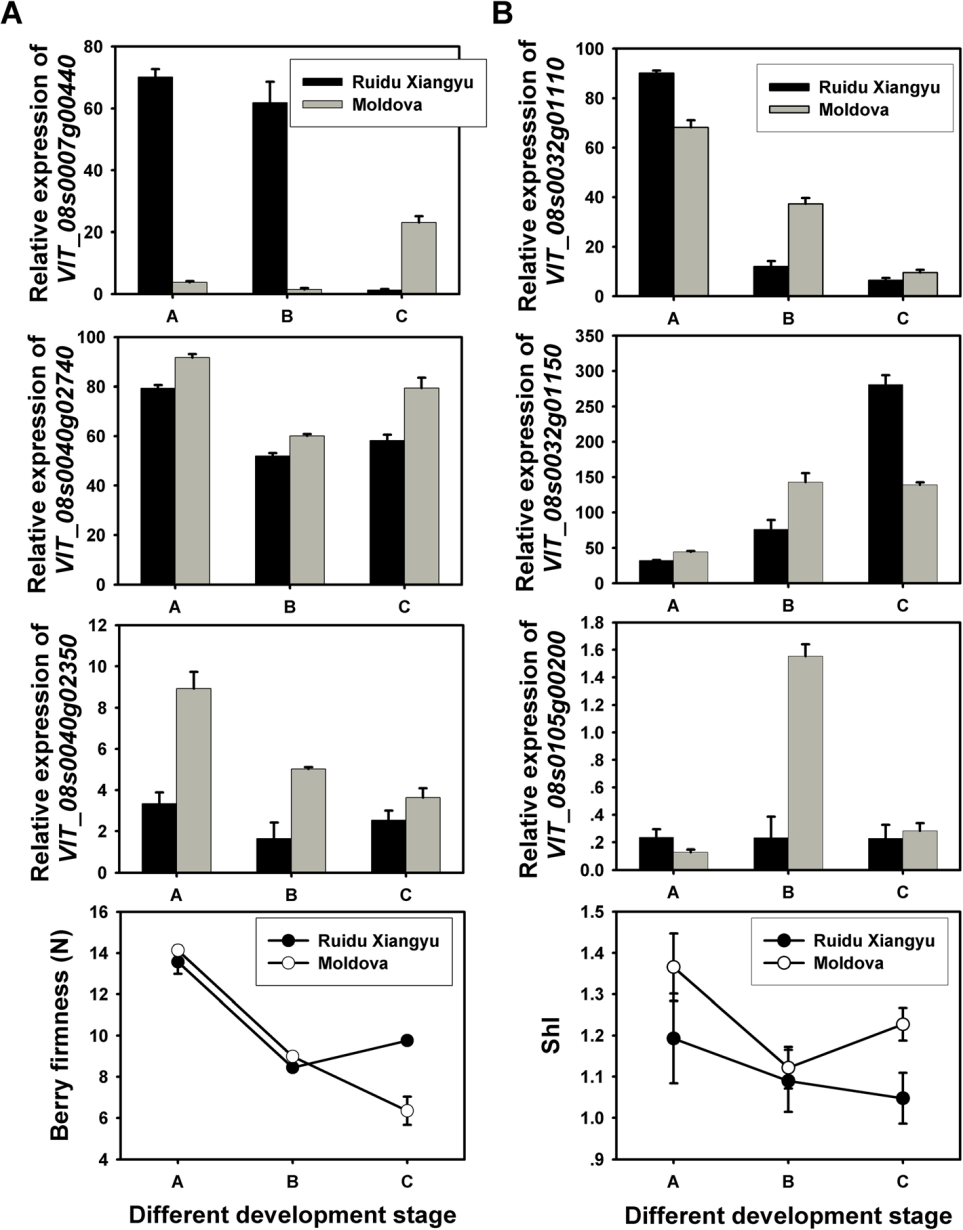
The Changes of berry firmness, berry shape index and expressions of candidate genes during grape berry development in ‘Moldova’ and ‘Ruidu Xiangyu’. **a** Expressions of three filtered candidate genes for berry firmness. **b** Expressions of three candidate genes for berry shape. Stage A: (young pea-size berries); Stage B: veraison (berries turning red or soft); Stage C: fully ripening stage (°Brix ≥18)

To further evaluate the potential relationship between candidate genes and each specific trait, the relative expression of corresponding candidate genes and berry related traits were analyzed during different grape berry development stages of two parent cultivars, ‘Moldova’ and ‘Ruidu Xiangyu’. As shown in Fig. 4a, berry firmness of ‘Moldova’ and ‘Ruidu Xiangyu’ both decreased at veraison. Thereafter, berry firmness of ‘Moldova’ still declined at the ripening stage, while that of ‘Ruidu Xiangyu’ increased significantly at the maturity stage (Fig. 4a). Among all the candidate genes (Supplementary Figure 8A), the expression pattern of *VIT_08s0040g02350* was consistent with that of parents berry firmness. There was an obvious increase in the expression of *VIT_08s0007g00440* in ‘Moldova’ at the ripening stage, while a significant decrease in ‘Ruidu Xiangyu’, which showed a contradictory pattern with the phenotypic variation.

The ShI of ‘Moldova’ and ‘Ruidu Xiangyu’ showed different change trends during berry development. Continuous reduction of ShI was observed in developing ‘Ruidu Xiangyu’, while ShI of ‘Moldova’ was firstly decreased but increased at the ripening stage. Among the analyzed genes, the relative expression of *VIT_08s0032g01110*, *VIT_08s0032g01150* and *VIT_08s0105g00200* showed a similar or opposite change patterns with that of ShI during berry development (Fig. 4b and Supplementary Figure 8B). In particular, the expression level of *VIT_08s0032g01150* was increased gradually during grape berry development stage in ‘Ruidu Xiangyu’, but in ‘Moldova’ the relative expression of *VIT_08s0032g01150* were increased at veraison and reduced at ripening stage, which presented a completely opposite trends with the changes of ShI (Fig. 4b).

Unfortunately, among all the candidate genes studied, no genes were found consistent with the changes of Muscat flavor in both cultivars during berry development (Supplementary Figure 8C).

### Overexpression of *VIT_08s0032g01110* in Arabidopsis

**Fig. 5:**
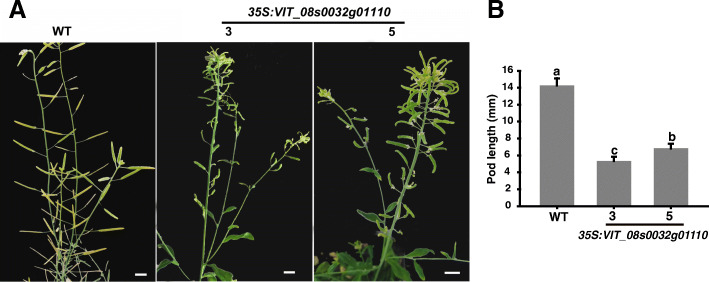
Pod shape and pod length of *35S:VIT08s0032g01110* and WT Arabidopsis plants. **a** Pod shape in *35S:VIT08s0032g01110* and WT plants; **b** Pod length of *35S:VIT08s0032g01110* and WT plants. Data are means from 20 pods. Bars are standard errors. Different letters indicate a statistical difference at *P* ≤ 0.05 among samples according to Duncan’ s multiple range test

Transgenic *Arabidopsis* plants overexpressing *VIT_08s0032g01110* were generated to elucidate its functions. As the results shown, differential pod shapes were observed between WT and *35S:VIT_08s0032g01110* seedlings (Fig. 5a). The pods of *35S:VIT_08s0032g01110* plants showed curved, and their lengths were shorter than WT plants (Fig. 5b).

## Discussion

### Genetic map

The construction of a genetic map is very essential for mining the genetic basis of relevant traits, in particular of high-density genetic map construction, which will improve the efficiency and accuracy of further QTL analysis [30]. The key step in the construction of high-density maps is the discovery and genotyping of molecular markers in high-throughput. The advent of NGS-based methods provides good opportunities for SNP markers development. Several high- density genetic maps for grapevine have been constructed with NGS techniques [7, 11, 33, 34, 40, 41]. In the present work, a high-density genetic map for ‘Moldova’ × ‘Ruidu Xiangyu’ was constructed with SLAF-seq technique. The final integrated genetic linkage map consisted of 3411 SLAF markers on 19 LGs spanning a total genetic distance of 3365.41 cM, with an average distance of 0.98 cM between adjacent markers (Table 2 and Supplementary Figure 2). Comparing to the previous reported maps constructed by the same SLAF-seq method [40, 41], the number of markers in this study is lower. This difference may be related to the F1 population used and markers filtering parameters in genetic map construction [7]. In addition, the percentage of “Gap < 5 cM” reached up to 97% (Table 2). But it needs to point out that total length of the maps in this study were over 3000 cM, which is similar to that of maps constructed by Zhu et al. [52], but longer than most of other published maps [7, 11, 25, 33, 34, 40, 41]. Collard et al. [53] have suggested that the difference in chromosome recombination events occurred during sexual reproduction in each subpopulation can be the reason for the variation in map length. It has been also suggested that the very large map length may probably result from the low quality of markers or difficulty in ordering abundant markers in small populations with low recombination [54]. We were not sure about the reasons for the large length of the maps constructed in this work. However, stringent filtering criteria were considered for the SLAF markers in this study to ensure the quality of marker. In the final, only 3411 markers from initial 96,416 polymorphic SLAF markers were grouped on the map. The average sequencing depths of these 3411 markers were up to 75.13-fold for ‘Ruidu Xiangyu’, 58.33-fold for ‘Moldova’, and 18.25-fold for each individual progeny (Supplementary Figure 3). Moreover, the low occurrence of double crossovers and deletion ratio were observed from haplotype maps (Supplementary Figure 4), indicating genotyping and marker-order was reliable in the LG. The heat map of the marker exchange relationship was generated for evaluating the linkage relationship among the markers, suggesting that the recombination frequency and mapping location between markers was basically consistent in each LG (Supplementary Figure 5). And a high level of genetic collinearity was observed between 19 LGs and the reference genome (Supplementary Figure 6). In short, the construction of this high- density map for ‘Moldova’ × ‘Ruidu Xiangyu’ provides a key foundation for genetic analysis of many agronomic traits in grapevine.

### QTL detection

The genetic factors for Muscat flavor have long been a concern of grape breeders. The researchers have identified a major QTL located on linkage group (LG) 5, and 1-deoxy-d-xylulose-5-phosphate synthase (*Vv*DXS) associated with the QTL has been considered as the candidate gene responsible for Muscat flavor [16–19]. In this work, four stable QTLs related to berry Muscat flavor were all mapped on LG5 (Table 3), which was consistent with the previous results [16–19]. A probable DXS gene (*VIT_05s0020g02130*) was found in the genomic region of 30.802–35.572 cM (Supplementary Table 9). While, It was noteworthy that *VIT_05s0020g03860* (a predicted homocysteine S-methyltransferase 3) was detected in the genomic region of 30.802–35.572 cM (Supplementary Table 9). Guo et al. [20] have investigated that *VIT_205s0020g03860* was significantly associated with berry flavor too. It has been suggested that Homocysteine S-methyltransferase catalyzing the inter-conversion of S-methylmethionine (SMM) and methionine (Met) is a part of the SMM cycle. The SMM cycle plays a fundamental role in plant metabolism [55]. But the specific function of Homocysteine S-methyltransferase gene in grapevine needs to be made further clear.

The berry firmness of F1 progenies and parents was evaluated by the compression test, which has been suggested as a reliable method for providing information about the firmness of whole unpeeled berries [56]. Good correlations have been investigated between the quantitative data obtained from the texture analyzer and sensory parameters made by tasting [22]. In our experimental population, the phenotype of firmness showed continuous variation. The fluctuation was observed in firmness across years, suggesting the effect of environmental factors on this trait. But relatively high broad sense heritability (*H*_*b*_^*2*^) of berry firmness was estimated reaching a value of 66.98% (Table 1), which were similar to the previous result (87.75% of *H*_*b*_^*2*^) obtained from ‘Ruby Seedless’ × ‘Sultanina’ progeny [23]. Based on the phenotypic data collected across 2 years, two major QTLs qBF-1 and qBF-2 were detected on LG 8 (Table 3). Correa et al. [23] have also identified a stable QTL for berry firmness across seasons on LG 8 in a progeny of ‘Ruby Seedless’ × ‘Sultanina’. Interestingly, we found an expansin-A4 (*VIT_08s0007g00440)* located at 14,732,840–14,734,936 base pairs (bp) and a probable pectate lyase 4 (*VIT_08s0040g02740*) located at 13,772,697–13,781,967 bp in the genomic region of the two major QTLs (Supplementary Table 9). During fruit ripening, changes in the cell wall structure affect fruit firmness [57], therefore the genes involved in cell wall formation and modification were considered to be the candidates. It has been suggested that expansins are involved in reassembly, degradation and expansion of cells and have a function in affecting berry softening in grape [58–60]. Pectate lyases have also been investigated to be related to cell wall composition [58, 61]. However, we did not identify QTLs on LG 1 and LG18, which have been reported in other populations [22, 25]. This may be due to different genetic backgrounds and distinct phenotype evaluation methods [25].

Fruit shape is one of the vital characteristic traits for horticulture crops. To date, various QTLs or candidate genes related to fruit shape have been genetically confirmed in different horticultural crops [62–66]. However, few genetic studies have focused on the identification of QTLs responsible for grape berry shape, although broad ranges of phenotypic variation in berry shape were observed. The grape berry shapes have always been intuitively classified by the traditional pictographic description, but it could not be directly used for QTL mapping analysis. Khambanonda [67]. has put forward the concept of “fruit shape index” to study the quantitative characters of pepper fruit. Fruit shape index can digitize the shape of fruit shape, which is more scientific than image description, and is more conducive to statistical analysis. Here, based on ShI data, qShI-1, qShI-2 and qShI-3 were identified on LG8 over 3 years (Table 3). In the genomic region of the QTLs, *VIT_08s0032g01110* (predicted axial regulator YABBY 5) and a cluster of E3 ubiquitin-protein ligase (*VIT_08s0105g00180*, *VIT_08s0105g00190*, *VIT_08s0105g00200* and *VIT_08s0105g00290*) were identified (Supplementary Table 9), which might play an important function in grape berry shape formation. Previous evidences from tomato have suggested that the FASCIATED locus of six QTLs influencing tomato fruit shape is encoded by a member of the YABBY family [63]. While, Song et al. [68] have investigated a QTL for rice grain width encodes a previously unknown RING-type E3 ubiquitin ligase. It is generally believed that homologous genes from different species may play similar biological functions [69].

### Validation of the candidate genes

It is suggested that candidate genes linked to a specific trait could play important role in molecular breeding. These functional genes could be transformed as molecular markers for using in MAS. For example, a single nucleotide polymorphism (SNP) within *VvDXS* (SNP1822 G > T) strongly associated with Muscat-flavored genotypes has been developed as DNA markers [70], which could be used in earlier stage selection of Muscat flavor breeding process. In this work, candidate genes were searched in QTL regions related the three berry quality traits. The expression profiles of several candidate genes were further analyzed. For Muscat flavor, no gene expression patterns showed similar to the changes of Muscat flavor in two parent cultivars including DXS (Supplementary Figure 8C) [19]. But this cannot exclude its role in Muscat flavor formation. Battilana et al. [71] have discovered the lysine with an asparagine at position 284 of the *Vv*DXS protein would affect Muscat flavor by influencing the enzyme catalytic efficiency. As to berry firmness, the expression of *VIT_08s0007g00440*, *VIT_08s0040g02740* and *VIT_08s0040g02350* showed consistent with the change of berry firmness (Fig. 4a), suggesting their possible role in affecting fruit firmness. Three candidate genes were highlighted for berry shape including *VIT_08s0032g01110*, *VIT_08s0032g01150* and *VIT_08s0105g00200*, their expression exhibited the similar or opposite change patterns with that of ShI during berry development (Fig. 4b). These genes might play positive or negative functions in grape berry shape formation. The changes in pods shape of transgenic Arabidopsis (Fig. 5) further confirmed the involvement of *VIT_08s0032g01110* in shape regulation. Now, validations of the other candidate genes in this work are still in progress. The applications of the candidate genes in future quality breeding could be expected in grapevine.

## Conclusions

In summary, a new high-density genetic map with total 3411 markers and an average distance of 0.98 cM between adjacent markers for ‘Moldova’ × ‘Ruidu Xiangyu’ was constructed with SLAF-seq technique, which provides a foundation for further genetic studies of relevant traits in grapevine. By using this map, 9 reliable QTLs linked to three grape berry quality traits were detected over 2–3 years. These QTL regions were significantly narrowed down compared to previous reports, which facilitated the subsequent candidate genes identification. The subsequent expression data of the candidate genes underlying the QTLs highlighted 3 genes related to berry firmness and 3 genes linked to berry shape respectively. Overexpression of *VIT_08s0032g01110* in transgenic Arabidopsis plants caused abnormal pod shape. These results broaden our knowledge of the genetic control of these berry related traits and give bases for further functional and efficient DNA markers development for MAS in grapevine quality breeding.

## Supplementary information

**Additional file 1: Table S1.** Primer sequences for candidate genes analyzed by real-time PCR. **Table S4** The information of the high-density paternal genetic map of male. **Table S5** The information of the high-density genetic map of female. **Table S6** The Spearman correlation coefficients between the genetic and physical positions of each linkage group on the integrated map. **Table S7** Summary of QTLs based on female map for three berry related traits over 3 successive years. **Table S8** Summary of QTLs based on male map for three berry related traits over 3 successive years.

**Additional file 2: Table S2** The phynotypic data of three berry related traits over 2–3 years.

**Additional file 3: Figure S1.** Number of markers in each of eight segregation patterns. For each segregation pattern, the left code of “×“represents the paternal genotype; the right code represents the maternal genotype. Such as for the segregation pattern of “ab × cd”, “ab” represents the paternal genotype, “cd” is the maternal genotype.

**Additional file 4: Table S3** List of genotyping data of 3411 SLAF markers among 160 F1 individuals.

**Additional file 5: Figure S2.** The consensus genetic linkage map generated using 160 hybrid seedlings derived from ‘Moldova’ × ‘Ruidu Xiangyu’.

**Additional file 6: Figure S3.** The average sequencing depths of markers on the consensus map in F1 population. The x-axis indicates individual F1 plant accessions; the y-axis indicates the average depths. The different letters (ac, ag, ak…) represents the distinct F1 progenies, “M” represents female parent and “P” represents the male parent.

**Additional file 7: Figure S4.** Haplotype maps of the consensus genetic map. Each row represents a SLAF marker, which is arranged in the order of position on the LG. Each two column represent the genotype of an F1 individual. The individual is separated by a blank column. The first column of each individual represents the paternal chromosome; the second column represents the maternal chromosome. The green represents the first allele from the parent; the blue represents the second allele from the parent, and the gray represents missing data.

**Additional file 8: Figure S5.** Heat maps of the paternal genetic map. Each cell represents the recombination rate of two markers. Yellow indicates a lower recombination rate and purple a higher one.

**Additional file 9: Figure S6.** Correlation of the genetic and physical positions. The x-axis represents the genetic groups; the y-axis represents the physical positions.

**Additional file 10: Figure S7.** Precise locations of major QTLs for berry Muscat flavor, firmness and berry shape on the parental maps. (A) LOD and PVE curves of QTL mapping for Muscat flavor on chromosome 5 of parental maps in 2016–2018; (B) LOD and PVE curves of QTL mapping for berry firmness on chromosome 8 of parental maps in 2017–2018; (C) LOD and PVE curves of QTL mapping for berry shape index on chromosome 8 of parental maps in 3 successive years. Short lines on x-axis indicate the genetic positions of the SLAF markers. Grey lines showed the threshold of LOD; Red lines represented the LOD value; Blue lines showed the PVE value.

**Additional file 11: Table S9** Candidate genes searched from the major QTL regions for three grape berry related traits.

**Additional file 12: Figure S8.** Heatmaps of berry firmness (BF), berry shape index (ShI), Muscat flavor (MF) and the expressions of candidate genes during grape berry development in ‘Moldova’ and ‘Ruidu Xiangyu’. (A) Expressions of all the filtered candidate genes for berry firmness. (B) Expressions of the filtered candidate genes for berry shape. (C) Expressions of candidate genes for berry Muscat flavor. Dark blue indicates a lower level and red a higher level. MA: “Moldova” at stage A; MB: “Moldova” at stage B; MC: “Moldova” at stage C; RA: “Ruidu Xiangyu” at stage A; MB: “Ruidu Xiangyu” at stage B; MC: “Ruidu Xiangyu” at stage C. Numbers in each colorful box represents the value of gene expression. Stage A: (young pea-size berries); Stage B: veraison (berries turning red or soft); Stage C: fully ripening stage (°Brix ≥18).

## Data Availability

The sequencing raw data in this study was deposited in the NCBI SRA database (Accession No: PRJNA657651, https://www.ncbi.nlm.nih.gov/sra/PRJNA657671). The *Vitis vinifera* reference genome referred in this work were downloaded from ftp://ftp.ensemblgenomes.org/pub/plants/release-25/fasta/vitis_vinifera/. The Phenotype data for Muscat flavor, berry firmness and berry shape in this work were presented in Supplementary Table 2. The information of molecular markers used for map construction in this work was listed in Supplementary Table 3. The genes were annotated and analyzed via the databases of Ensembl Plants (http://plants.ensembl.org/index.html) and NCBI (https://www.ncbi.nlm.nih.gov/). The primers for qRT-PCR used in this research were designed by the Primer-Blast from NCBI (https://www.ncbi.nlm.nih.gov/tools/primerblast/) and their sequences are listed in Supplementary Table 1. Arabidopsis plants used in this research were treated following the guidelines of ABRC.

## Abbreviations

QTL: Quantitative trait locus
LG: Linkage group
SLAF: Specific length amplified fragment
MAS: Marker assisted selection
DXS: 1-deoxy-d-xylulose-5-phosphate synthase
SNP: Single nucleotide polymorphisms
AFLP: Amplified fragment length polymorphism
SRAP: Sequence related amplified polymorphism
SSR: Single sequence repeat
NGS: Next generation sequencing
ShI: Berry shape index
BL: Berry length
BD: Berry diameter
*H*_*b*_^*2*^: Broad sense heritability
CV: Coefficient of variation
MLOD: Modified logarithm of odds
MF: Muscat flavor
BF: Berry firmness
PVE: Phenotypic variance explained by individual QTL
