# Supplementary material for: Novel stable QTLs identification for berry quality traits based on high-density genetic linkage map construction in table grape
Source: BMC Plant Biol. 2020 Sep 3;20:411. doi: 10.1186/s12870-020-02630-x (PMC7470616; doi:10.1186/s12870-020-02630-x)
Supplement: Supplementary file 1 — Additional file 1: Table S1. Primer sequences for candidate genes analyzed by real-time PCR. Table S4 The information of the high-density paternal genetic map of male. Table S5 The information of the high-density genetic map of female. Table S6 The Spearman correlation coefficients between the genetic and physical positions of each linkage group on the integrated map. Table S7 Summary of QTLs based on female map for three berry related traits over 3 successive years. Table S8 Summary of QTLs based on male map for three berry related traits over 3 successive years. [file 12870_2020_2630_MOESM1_ESM.docx]

Supplementary Table 1.Primer sequences for candidate genes analyzed by real-time PCR.

| **Gene ID** | **Primer sequences** |
| --- | --- |
| *VIT_08s0032g01110* | F: CCATTAGGTGTGGCCACTGT; R: GCTGCACCTTTTGGAAGCAT |
| *VIT_08s0032g00920* | F: GGTGGAAGGAGAGGCTGTTC; R: GCAGCTGGTGTATTGTTGATAC |
| *VIT_08s0032g01080* | F: TGCGAAGTATTTGGCTAACTTGT; R: ATGCCAAACCCACACAGGAT |
| *VIT_08s0032g01090* | F: TGTTGACACTATGTGTGCATTTTCA; R: GGTTGTCTGCCCTGAGAAGT |
| *VIT_08s0032g01150* | F: TCGCTCTCAAGAAGGCCAAG; R: TCGCGATACTCGGAGGAGAT |
| *VIT_08s0032g01180* | F: TTGGAGGCCGAACAGTGAAG; R: CTCTAAGCCCCTCCGATGTG |
| *VIT_08s0105g00180* | F: CGGTGTCAACGGGTATAACG; R: AAGCATGGAGAGTACACCAC |
| *VIT_08s0105g00190* | F: CGGTGTCAACGGGTATAACG; R: AAGCATTGAGAGTGCACCA |
| *VIT_08s0105g00200* | F: CGAGTGTTGTAGGGGTGCTT; R: CATCAGCGCAGGAGCATAGA |
| *VIT_08s0105g00290* | F: TCATGCAGCGTCATCACAGT; R: GCACCACGATAATGCACGAC |
| *VIT_08s0217g00020* | F: CTTGCCAGTGACGAGACAGT; R: GCCTTACAAGCCTCAGAGCA |
| *VIT_08s0007g00440* | F: ATTGAACGTAGCGGGTTGGT; R: GCCGGTTTTCACTTTCACCC |
| *VIT_08s0040g02740* | F: TCCATCAAACACAGTCCGCA; R: GGTCCGTGAAGACCACCAAT |
| *VIT_08s0040g02340* | F: CCTTGCGCTCAGTCACATTG; R: CAGCGTGAGGGATAAGGAAT |
| *VIT_08s0040g02350* | F: ATCCAGAAAGATGGGGTCGC; R: TTGGTATCGTCCCCACTGGA |
| *VIT_08s0007g00600* | F: TTTCACCTGTGATGGGTCCG; R: TGACAAACCAGGGGGGAGGA |
| *VIT_08s0007g00660* | F: AGAGAGCCACCCACTTCATC; R: CGCTTCCCATCCGTGACA |
| *VIT_08s0007g00670* | F: ATCTCACTCCCCTAACGCCA; R: GAATGTGGGAAGTGCCGGTC |
| *VIT_08s0007g00680* | F: TACTGTCAACGGGCAGTTCC; R: AAGCCAGAACCCATCTCAGC |
| *VIT_08s0007g00690* | F: CTTTGACACCGACACGCTTC; R: GGCAATGCCGGTTTGAAGAA |
| *VIT_08s0007g01550* | F: CCATGTGCAAAACCTGACCG; R: TAGTTGGAACACCCCATGCC |
| *VIT_05s0020g01240* | F: ACCATGAGTTACAGAACTGGCA; R: CTCTAACAGCGGTCCAGCTT |
| *VIT_05s0020g02130* | F: CTTGGACAAACAAGGGAGGC; R: CACTTTCAAGCCTTATTGCTGAC |
| *VIT_05s0020g01840* | F: ATTCATCAACCAGAATCAGAGGT; R: TGTACCCAGTTGAACCCACC |
| *VIT_05s0020g03860* | F: TCAACTGCACCCCTCCTAGA; R: TCACGCCACTTGCTCACATA |
| *VIT_05s0020g03640* | F: AATCGAGCACTACCACCACC; R: GTTGGCATCCTTTCGGCATC |
| *VIT_05s0020g03170* | F: TTGGCCAGGCACATTCTCAT; R: GGGTCCTCAACTGCTACACC |
| *VvGAPDH* | F: TTCTCGTTGAGGGCTATTCCA; R: CCACAGACTTCATCGGTGACA |
| *VvUbiquitin* | F: GTGGTATTATTGAGCCATCCTT; R: AACCTCCAATCCAGTCATCTAC |

Supplementary Table 4 The information of the high-density paternal genetic map of male

| **LG ID** | **No of SLAFs** | **Distance (cM)** | **Average distance between markers (cM)** | **Largest gap** | **Gap˂5cM** |
| --- | --- | --- | --- | --- | --- |
| LG1 | 94 | 183.31 | 1.95 | 34.61 | 95% |
| LG2 | 120 | 165.73 | 1.38 | 17.83 | 96% |
| LG3 | 87 | 186.04 | 2.14 | 26.23 | 89% |
| LG4 | 68 | 148.2 | 2.18 | 26.59 | 93% |
| LG5 | 73 | 146.14 | 2.00 | 25.54 | 92% |
| LG6 | 141 | 217.18 | 1.54 | 15.23 | 96% |
| LG7 | 124 | 154.74 | 1.25 | 18.73 | 93% |
| LG8 | 130 | 178.73 | 1.37 | 42.78 | 93% |
| LG9 | 129 | 135.7 | 1.05 | 8.03 | 95% |
| LG10 | 113 | 222.12 | 1.97 | 20.59 | 97% |
| LG11 | 61 | 170.74 | 2.80 | 41.33 | 88% |
| LG12 | 159 | 187.25 | 1.18 | 16.08 | 96% |
| LG13 | 188 | 199.79 | 1.06 | 20.78 | 94% |
| LG14 | 176 | 196.47 | 1.12 | 19.65 | 93% |
| LG15 | 47 | 164.81 | 3.51 | 39.26 | 86% |
| LG16 | 79 | 163.97 | 2.08 | 26.44 | 93% |
| LG17 | 126 | 189.02 | 1.50 | 15.22 | 98% |
| LG18 | 101 | 168.56 | 1.67 | 32.22 | 98% |
| LG19 | 118 | 164.25 | 1.39 | 12.74 | 97% |
| Total | 2134 | 3,342.75 | 1.57 | / | 94% |

Supplementary Table 5 The information of the high-density genetic map of female

| **LG ID** | **No of SLAFs** | **Distance (cM)** | **Average distance between markers (cM)** | **Largest gap** | **Gap˂5cM** |
| --- | --- | --- | --- | --- | --- |
| LG1 | 91 | 197.9 | 2.17 | 11.16 | 90% |
| LG2 | 48 | 106.63 | 2.22 | 17.07 | 82% |
| LG3 | 105 | 199.42 | 1.90 | 23.50 | 90% |
| LG4 | 85 | 137.93 | 1.62 | 14.38 | 90% |
| LG5 | 100 | 119.84 | 1.20 | 19.65 | 93% |
| LG6 | 112 | 142.79 | 1.27 | 15.23 | 93% |
| LG7 | 60 | 201.05 | 3.35 | 33.43 | 86% |
| LG8 | 134 | 180.97 | 1.35 | 21.54 | 95% |
| LG9 | 129 | 201.98 | 1.57 | 33.42 | 95% |
| LG10 | 83 | 112.74 | 1.36 | 35.92 | 93% |
| LG11 | 55 | 144.38 | 2.63 | 22.51 | 83% |
| LG12 | 114 | 161.79 | 1.42 | 30.00 | 97% |
| LG13 | 91 | 185.96 | 2.04 | 20.84 | 93% |
| LG14 | 159 | 172.39 | 1.08 | 11.94 | 94% |
| LG15 | 88 | 168.28 | 1.91 | 16.28 | 92% |
| LG16 | 84 | 118.43 | 1.41 | 20.59 | 93% |
| LG17 | 65 | 103.89 | 1.60 | 33.42 | 92% |
| LG18 | 125 | 181.34 | 1.45 | 18.74 | 94% |
| LG19 | 120 | 181.19 | 1.51 | 19.65 | 92% |
| Total | 1848 | 3,018.90 | 1.63 | / | 92% |

Supplementary Table 6 The Spearman correlation coefficients between the genetic and physical positions of each linkage group on the integrated map.

| Linkage group ID | Spearman | Linkage group ID | Spearman |
| --- | --- | --- | --- |
| Chr1  Chr2  Chr3  Chr4  Chr5  Chr6  Chr7  Chr8  Chr9  Chr10 | 0.97  0.95  0.88  0.87  0.93  0.92  0.97  0.85  0.96  0.95 | Chr11  Chr12  Chr13  Chr14  Chr15  Chr16  Chr17  Chr18  Chr19 | 0.92  0.96  0.98  0.87  0.89  0.91  0.95  0.97  0.96 |

Supplementary Table 7 Summary of QTLs based on female map for three berry related traits over 3 successive years

| **Trait** | **QTL** | | **Chr** | **Year of detection** | **Flanking Markers** | **Interval (cM)** | **Maximum**  **LOD** | **PVE**  **(%)** |
| --- | --- | --- | --- | --- | --- | --- | --- | --- |
| MF | qMF-1 | | 5 | 2016 | Marker2668298-  Marker2860950 | 5.420-10.452 | 3.38 | 13.70 |
|  | qMF-2 | | 5 | 2016 | Marker2793749- Marker2154785 | 13.628-16.789 | 4.18 | 16.60 |
|  | qMF-3 | | 5 | 2017 | Marker2674097- Marker2853250 | 2.896-17.418 | 6.75 | 24.0 |
|  | qMF-4 | | 5 | 2018 | Marker2761785- Marker2861066 | 0.629-18.684 | 3.75 | 16.90 |
| BF | qBF-1 | | 8 | 2017 | Marker1446374-Marker1505260 | 135.267-141.629 | 4.11 | 19.40 |
|  | qBF-2 | | 8 | 2018 | Marker1446374-Marker1442546 | 135.267-141.000 | 3.28 | 20.0 |
| ShI | qShI-1 | | 8 | 2016 | Marker1415438-Marker1450563 | 2.565-8.952 | 6.34 | 22.0 |
|  | qShI-2 | | 8 | 2016 | Marker1399465 | 38.985 | 4.57 | 16.20 |
|  | qShI-3 | 8 | | 2017 | Marker1415438- Marker1563052 | 2.565-8.952 | 5.44 | 19.20 |
|  | qShI-4 | | 8 | 2018 | Marker1507731- Marker1456093 | 2.565-59.783 | 5.82 | 26.0 |

Note: Chr indicates chromosome; LOD indicates the logarithm of odds score; PVE indicates the phenotypic variance explained by individual QTL; MF is abbreviation of Muscat flavor; BF is abbreviation of berry firmness; ShI represents berry shape index.

Supplementary Table 8 Summary of QTLs based on male map for three berry related traits over 3 successive years

| **Trait** | **QTL** | | **Chr** | **Year of detection** | **Flanking Markers** | **Interval (cM)** | **Maximum**  **LOD** | **PVE**  **(%)** |
| --- | --- | --- | --- | --- | --- | --- | --- | --- |
| MF | qMF-1 | | 5 | 2016 | Marker2746921-  Marker2755502 | 30.013-33.836 | 3.51 | 14.20 |
|  | qMF-2 | | 5 | 2016 | Marker2674746-  Marker2782663 | 41.456-43.960 | 4.17 | 16.40 |
|  | qMF-3 | | 5 | 2017 | Marker2746921-  Marker2782663 | 30.013-43.960 | 6.11 | 21.50 |
|  | qMF-4 | | 5 | 2018 | Marker2759265-  Marker2782663 | 27.248-43.960 | 3.76 | 19.40 |
| BF | qBF-1 | | 8 | 2017 | Marker1558508-Marker1505260 | 160.891-162.149 | 4.14 | 19.0 |
|  | qBF-2 | | 8 | 2018 | Marker1558508-Marker1505260 | 160.891-162.149 | 3.34 | 20.10 |
| ShI | qShI-1 | | 8 | 2016 | Marker1416154-Marker1434785 | 0-3.227 | 5.65 | 19.20 |
|  | qShI-2 | | 8 | 2016 | nd | nd | nd | nd |
|  | qShI-3 | | 8 | 2017 | Marker1416154-Marker1434785 | 0-3.227 | 5.21 | 19.10 |
|  | qShI-4 | 8 | | 2018 | Marker1434785-  Marker1474867 | 3.227-7.126 | 5.65 | 23.7 |

Note: Chr indicates chromosome; LOD indicates the logarithm of odds score; PVE indicates the phenotypic variance explained by individual QTL; MF is abbreviation of Muscat flavor; BF is abbreviation of berry firmness; ShI represents berry shape index; nd represents no significant QTL was detected.
