# Supplementary figures and images for: Novel stable QTLs identification for berry quality traits based on high-density genetic linkage map construction in table grape
Source: BMC Plant Biol. 2020 Sep 3;20:411. doi: 10.1186/s12870-020-02630-x (PMC7470616; doi:10.1186/s12870-020-02630-x)

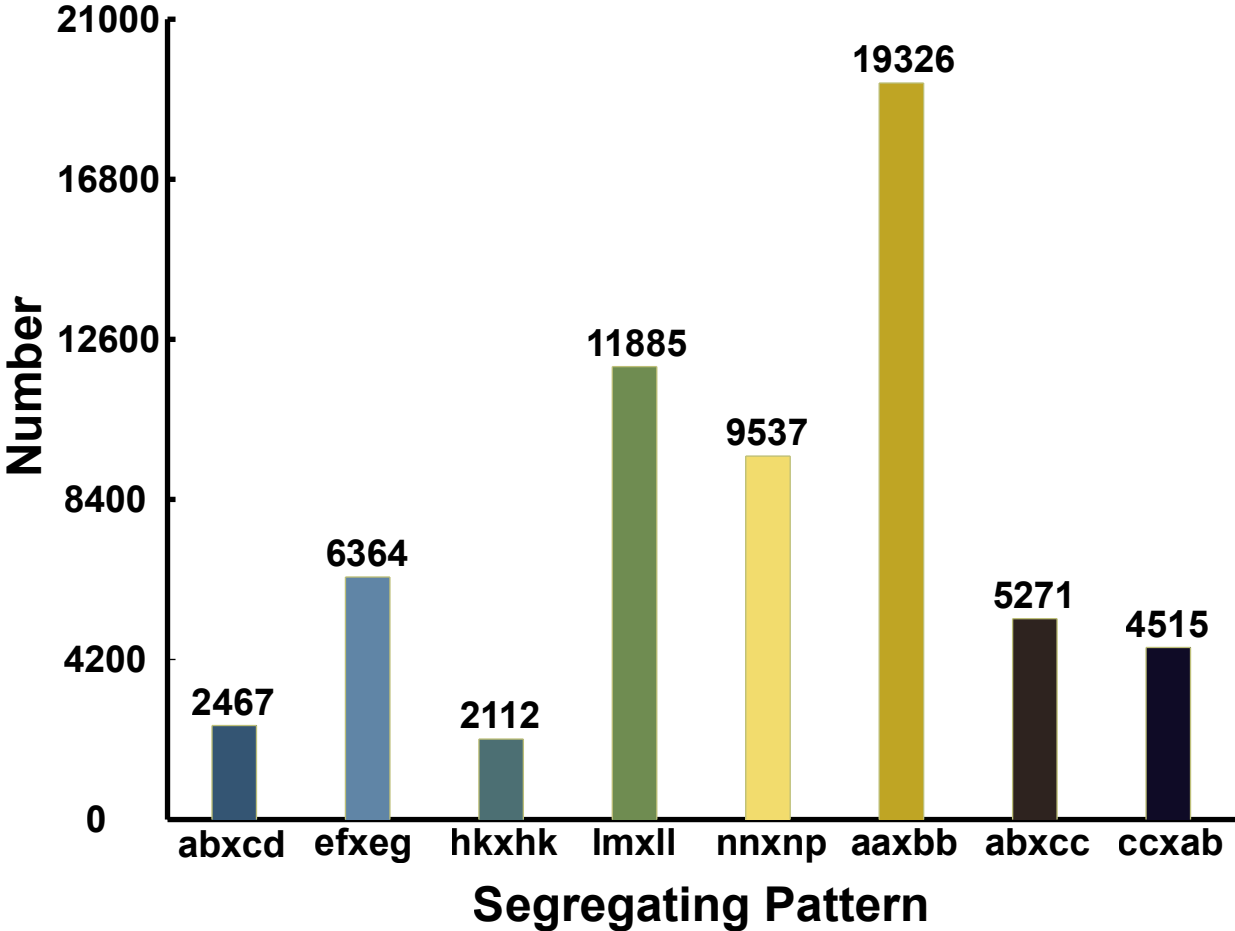

Supplement: Supplementary file 3 — Additional file 3: Figure S1. Number of markers in each of eight segregation patterns. For each segregation pattern, the left code of “×“represents the paternal genotype; the right code represents the maternal genotype. Such as for the segregation pattern of “ab × cd”, “ab” represents the paternal genotype, “cd” is the maternal genotype. [file 12870_2020_2630_MOESM3_ESM.pdf]

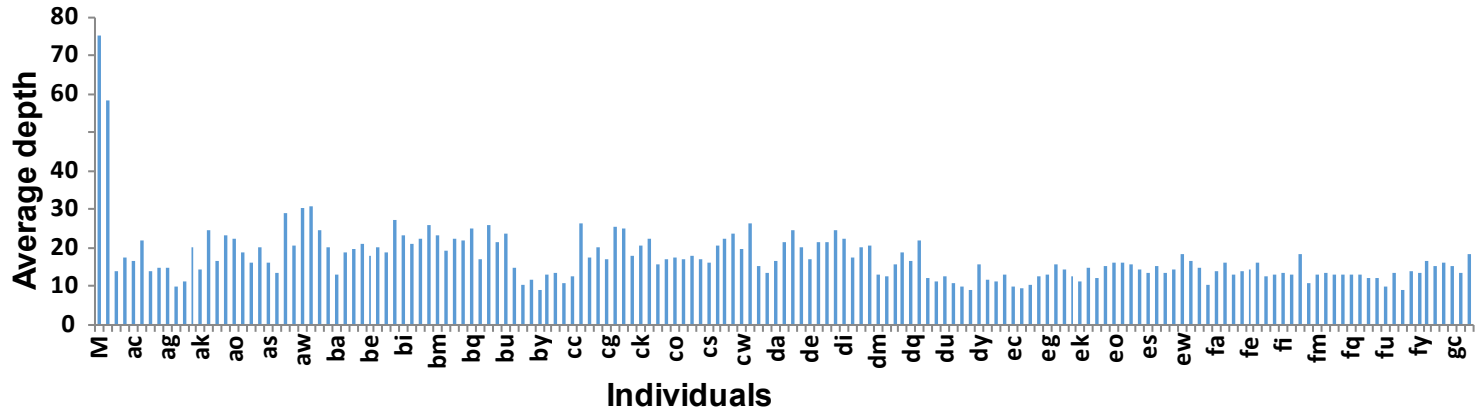

Supplement: Supplementary file 6 — Additional file 6: Figure S3. The average sequencing depths of markers on the consensus map in F1 population. The x-axis indicates individual F1 plant accessions; the y-axis indicates the average depths. The different letters (ac, ag, ak…) represents the distinct F1 progenies, “M” represents female parent and “P” represents the male parent. [file 12870_2020_2630_MOESM6_ESM.pdf]

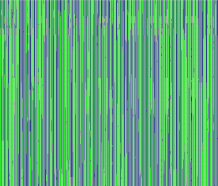

**LG1**

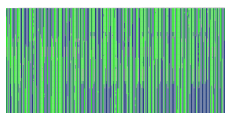

**LG2**

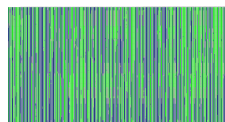

**LG3**

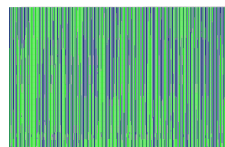

**LG4**

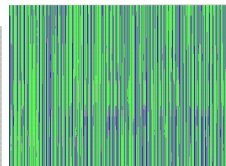

**LG5**

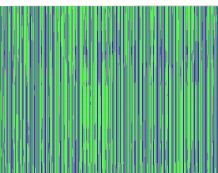

**LG6**

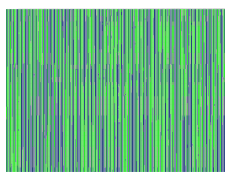

**LG7**

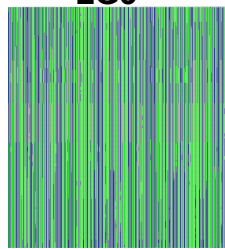

**LG8**

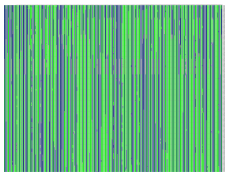

**LG9**

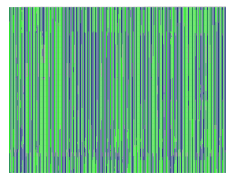

**LG10**

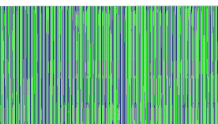

**LG11**

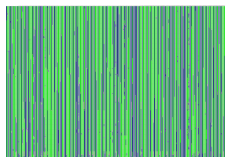

**LG12**

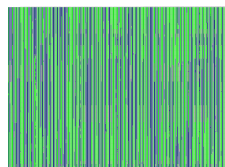

**LG13**

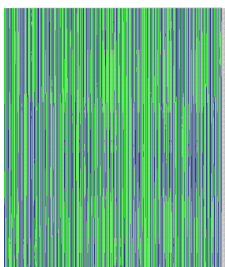

**LG14**

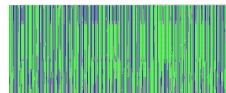

**LG15**

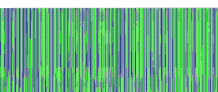

**LG16**

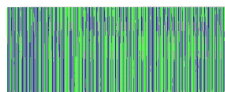

**LG17**

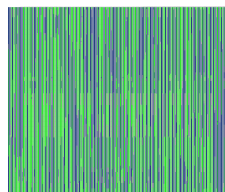

**LG18**

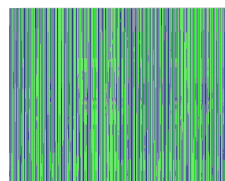

**LG19**

Supplement: Supplementary file 7 — Additional file 7: Figure S4. Haplotype maps of the consensus genetic map. Each row represents a SLAF marker, which is arranged in the order of position on the LG. Each two column represent the genotype of an F1 individual. The individual is separated by a blank column. The first column of each individual represents the paternal chromosome; the second column represents the maternal chromosome. The green represents the first allele from the parent; the blue represents the second allele from the parent, and the gray represents missing data. [file 12870_2020_2630_MOESM7_ESM.pdf]

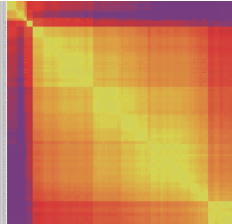

**LG1**

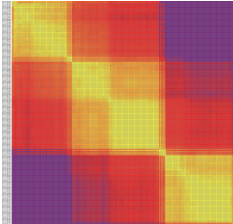

**LG2**

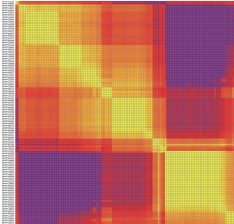

**LG3**

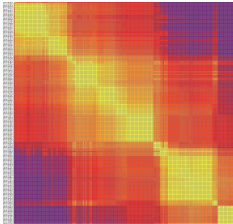

**LG4**

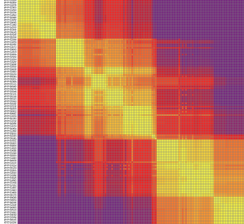

**LG5**

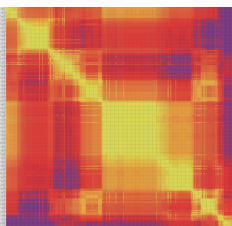

**LG6**

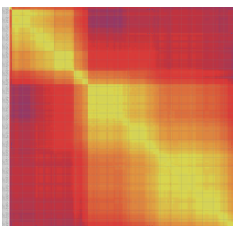

**LG7**

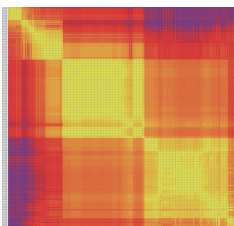

**LG8**

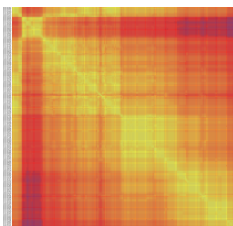

**LG9**

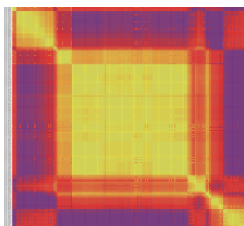

**LG10**

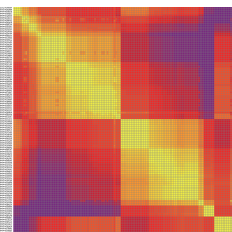

**LG11**

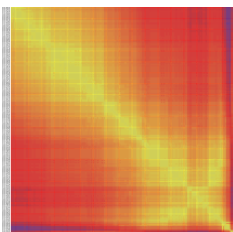

**LG12**

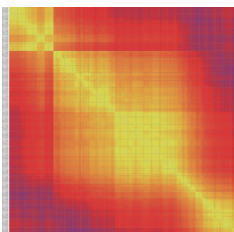

**LG13**

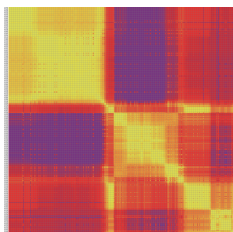

**LG14**

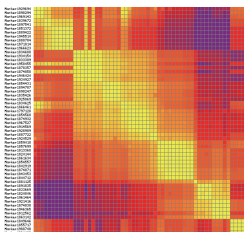

**LG15**

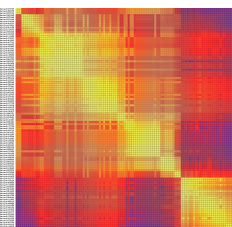

**LG16**

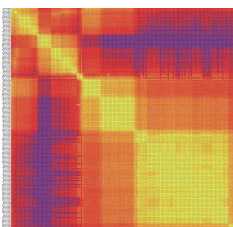

**LG17**

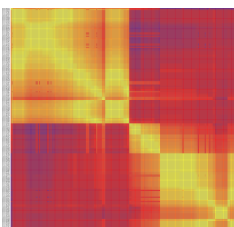

**LG18**

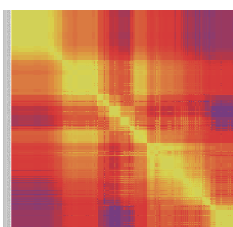

**LG19**

Supplement: Supplementary file 8 — Additional file 8: Figure S5. Heat maps of the paternal genetic map. Each cell represents the recombination rate of two markers. Yellow indicates a lower recombination rate and purple a higher one. [file 12870_2020_2630_MOESM8_ESM.pdf]

genome

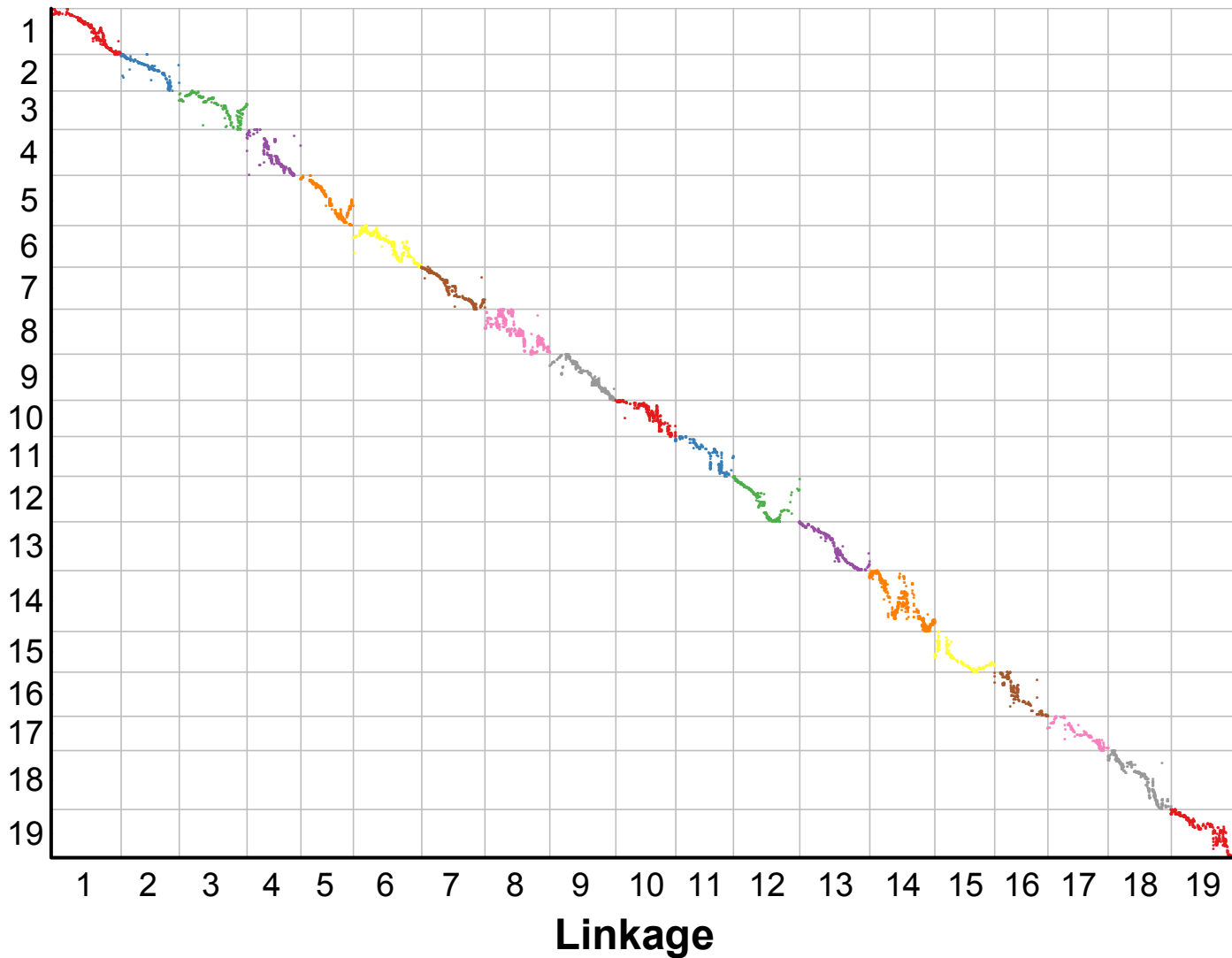

Supplement: Supplementary file 9 — Additional file 9: Figure S6. Correlation of the genetic and physical positions. The x-axis represents the genetic groups; the y-axis represents the physical positions. [file 12870_2020_2630_MOESM9_ESM.pdf]

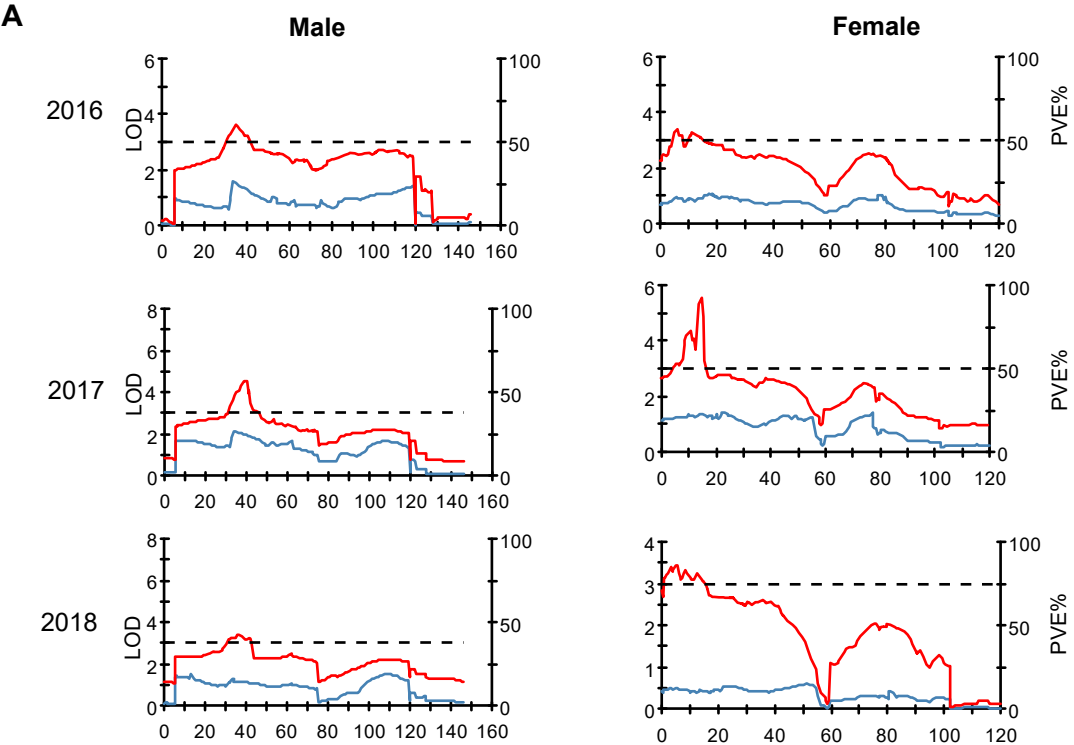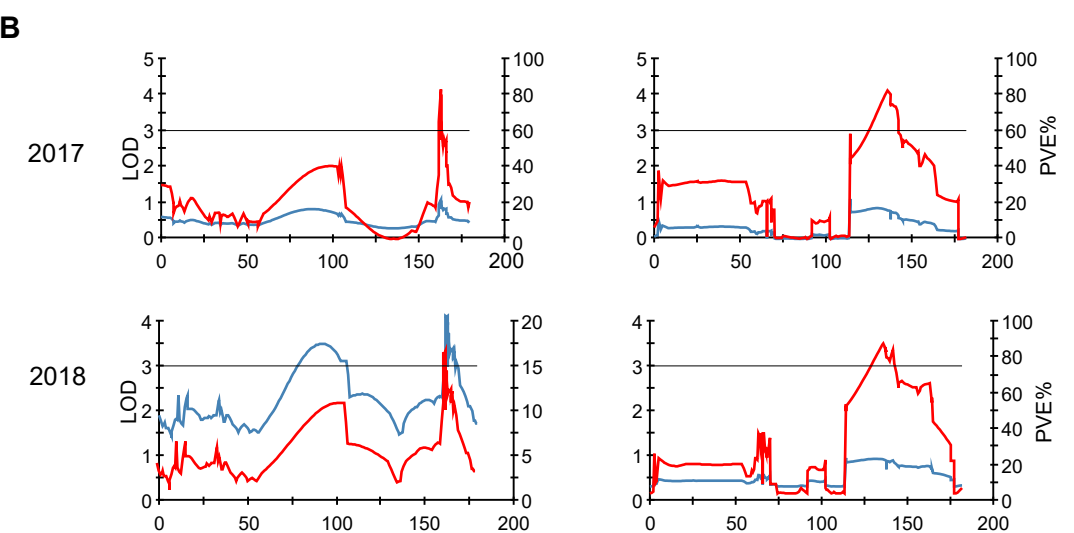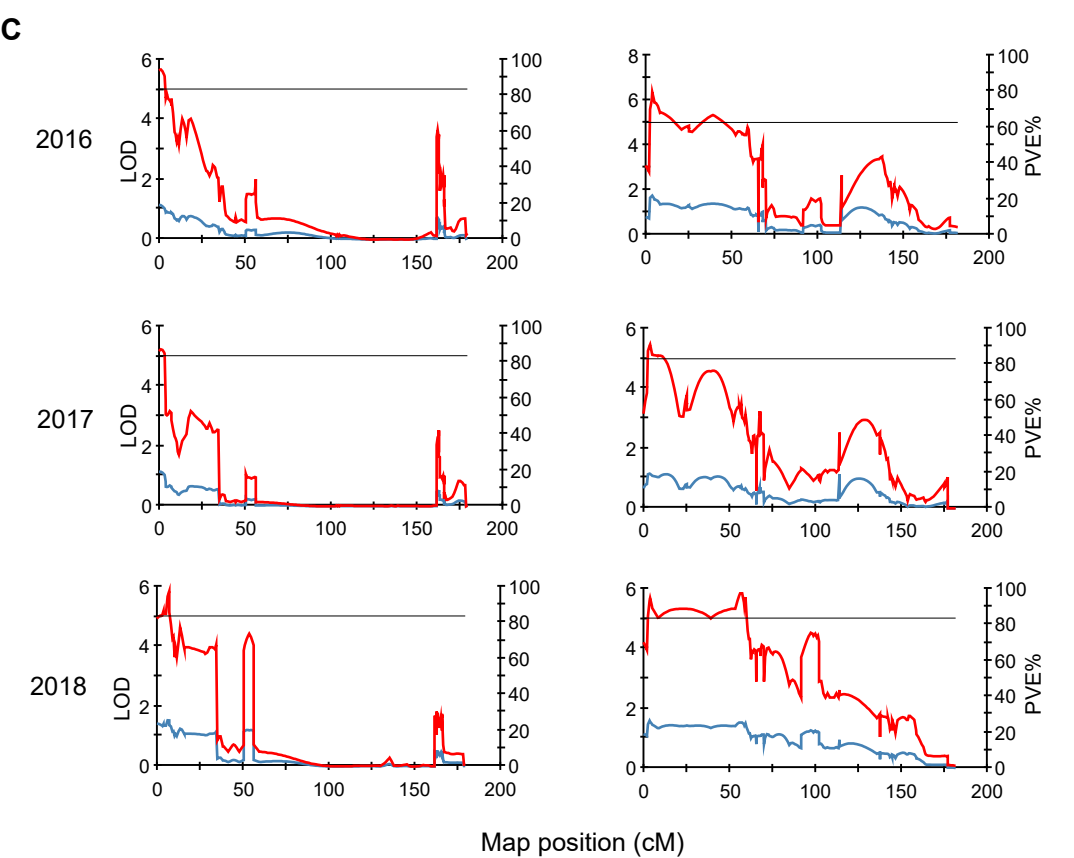

Supplement: Supplementary file 10 — Additional file 10: Figure S7. Precise locations of major QTLs for berry Muscat flavor, firmness and berry shape on the parental maps. (A) LOD and PVE curves of QTL mapping for Muscat flavor on chromosome 5 of parental maps in 2016–2018; (B) LOD and PVE curves of QTL mapping for berry firmness on chromosome 8 of parental maps in 2017–2018; (C) LOD and PVE curves of QTL mapping for berry shape index on chromosome 8 of parental maps in 3 successive years. Short lines on x-axis indicate the genetic positions of the SLAF markers. Grey lines showed the threshold of LOD; Red lines represented the LOD value; Blue lines showed the PVE value. [file 12870_2020_2630_MOESM10_ESM.pdf]
