# Supplementary material for: Novel stable QTLs identification for berry quality traits based on high-density genetic linkage map construction in table grape
Source: BMC Plant Biol. 2020 Sep 3;20:411. doi: 10.1186/s12870-020-02630-x (PMC7470616; doi:10.1186/s12870-020-02630-x)
Supplement: Supplementary file 12 — Additional file 12: Figure S8. Heatmaps of berry firmness (BF), berry shape index (ShI), Muscat flavor (MF) and the expressions of candidate genes during grape berry development in ‘Moldova’ and ‘Ruidu Xiangyu’. (A) Expressions of all the filtered candidate genes for berry firmness. (B) Expressions of the filtered candidate genes for berry shape. (C) Expressions of candidate genes for berry Muscat flavor. Dark blue indicates a lower level and red a higher level. MA: “Moldova” at stage A; MB: “Moldova” at stage B; MC: “Moldova” at stage C; RA: “Ruidu Xiangyu” at stage A; MB: “Ruidu Xiangyu” at stage B; MC: “Ruidu Xiangyu” at stage C. Numbers in each colorful box represents the value of gene expression. Stage A: (young pea-size berries); Stage B: veraison (berries turning red or soft); Stage C: fully ripening stage (°Brix ≥18). [file 12870_2020_2630_MOESM12_ESM.pdf]

A

	MA	MB	MC	RA	RB	RC
VIT_08s0007g00440	3.77	1.49	23.04	70.10	61.86	1.30
VIT_08s0040g02740	91.72	60.04	79.51	79.33	51.92	58.25
VIT_08s0040g02340	8.83	5.21	3.55	3.47	1.89	1.85
VIT_08s0040g02350	8.92	5.03	3.63	3.34	1.64	2.54
VIT_08s0007g00600	1.28	0.06	0.05	1.18	0.13	0.01
VIT_08s0007g00660	0.57	0.02	0.00	0.86	0.02	0.02
VIT_08s0007g00670	0.42	0.01	0.00	1.41	0.01	0.00
VIT_08s0007g00680	0.70	0.06	0.02	1.46	0.11	0.01
VIT_08s0007g00690	6.82	0.40	0.00	2.54	2.68	0.06
VIT_08s0007g01550	0.04	0.11	0.02	0.26	0.09	0.00
**BF**	14.13	8.99	6.35	13.56	8.45	9.76

B

	MA	MB	MC	RA	RB	RC
VIT_08s0032g01110	68.23	37.26	9.56	90.08	12.00	6.44
VIT_08s0032g00920	93.47	64.25	59.41	50.88	37.58	67.19
VIT_08s0032g01080	6.21	6.25	6.10	3.90	3.53	5.52
VIT_08s0032g01090	8.62	8.51	9.07	5.84	4.59	12.34
VIT_08s0032g01150	44.37	142.86	138.85	32.36	75.99	280.80
VIT_08s0032g01180	25.19	17.05	7.48	18.74	9.92	7.67
VIT_08s0105g00180	5.04	23.72	8.81	1.85	2.44	1.17
VIT_08s0105g00190	3.33	16.72	6.42	2.23	3.63	2.18
VIT_08s0105g00200	0.13	1.55	0.28	0.24	0.23	0.23
VIT_08s0105g00290	27.27	28.17	20.30	25.47	21.68	16.48
VIT_08s0217g00020	90.18	124.60	69.90	100.69	62.54	72.70
**ShI**	1.37	1.12	1.23	1.19	1.09	1.05

C

	MA	MB	MC	RA	RB	RC
VIT_05s0020g01240	13.06	8.02	0.74	6.94	1.19	0.57
VIT_05s0020g02130	94.62	107.50	26.15	133.95	32.12	15.65
VIT_05s0020g01840	6.31	7.88	2.22	17.28	3.19	2.33
VIT_05s0020g03860	3.11	57.11	127.60	1.37	304.66	288.04
VIT_05s0020g03640	1.37	1.99	0.01	6.71	0.35	0.04
VIT_05s0020g03170	10.67	10.97	7.48	58.15	11.73	1.30
**MF**	0.00	0.00	0.00	0.00	0.00	4.32

Minimum **Maximum**
