# Supplementary material for: Novel stable QTLs identification for berry quality traits based on high-density genetic linkage map construction in table grape
Source: BMC Plant Biol. 2020 Sep 3;20:411. doi: 10.1186/s12870-020-02630-x (PMC7470616; doi:10.1186/s12870-020-02630-x)
Supplement: Supplementary file 5 — Additional file 5: Figure S2. The consensus genetic linkage map generated using 160 hybrid seedlings derived from ‘Moldova’ × ‘Ruidu Xiangyu’. [file 12870_2020_2630_MOESM5_ESM.pdf]

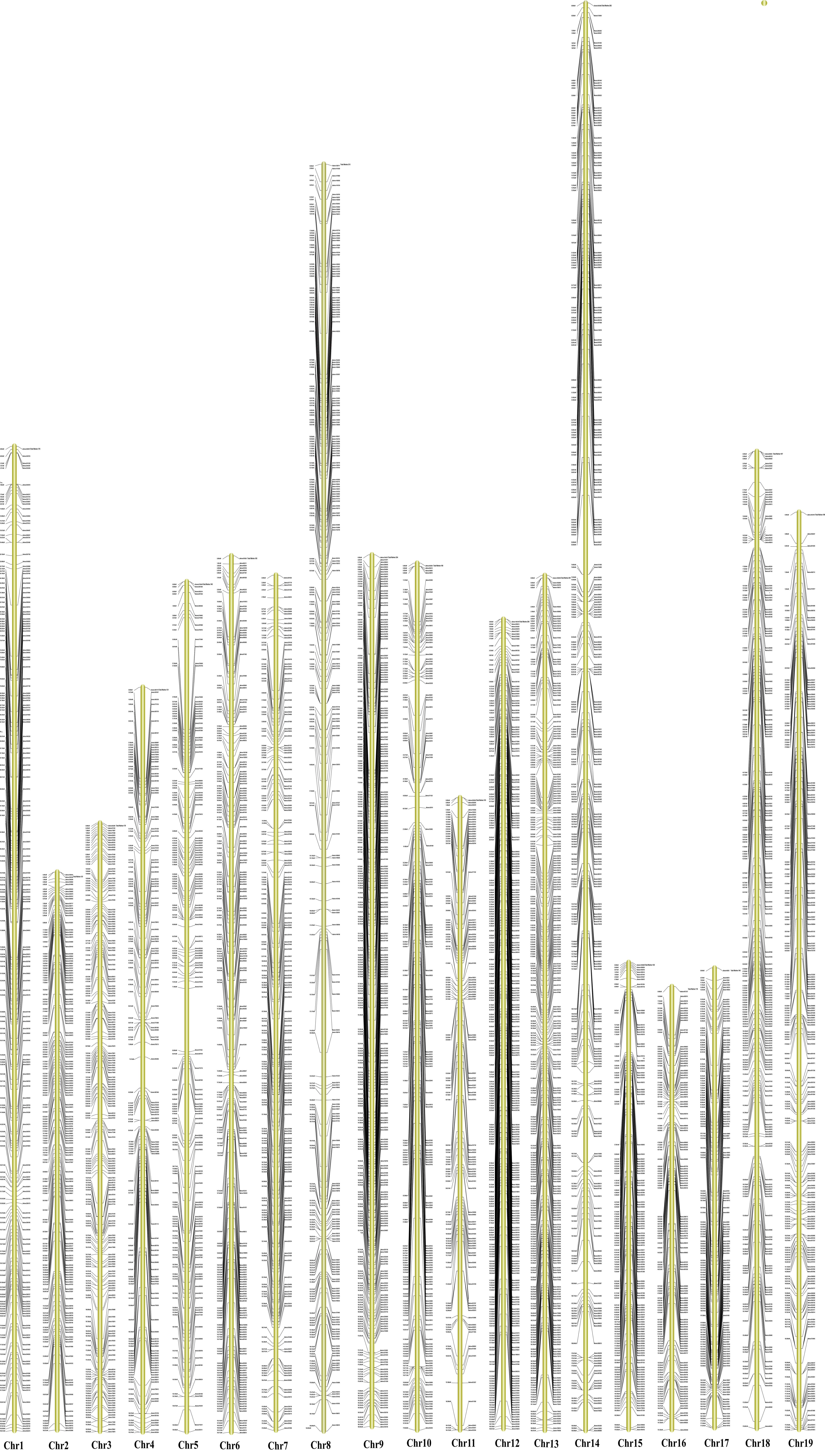
Chr1
Chr2
Chr3
Chr4
Chr5
Chr6
Chr7
Chr8
Chr9
Chr10
Chr11
Chr12
Chr13
Chr14
Chr15
Chr16
Chr17
Chr18
Chr19
